# Interleukin-6 in synovial fluid drives the conversion of DC2s to DC3s in inflammatory arthritis

**DOI:** 10.1016/j.isci.2025.112957

**Published:** 2025-06-20

**Authors:** Annika H. Decker, Lucas L. van den Hoogen, Tom van Oorschot, Liliana Sanchez-Rocha, Martine A. Boks, Ghaith Bakdash, Ranjeny Thomas, Calin D. Popa, Martijn Verdoes, Rogier M. Thurlings, Anouk M.D. Becker, I. Jolanda M. de Vries

**Affiliations:** 1 Medical BioSciences, Radboud University Medical Center, 6525 GA Nijmegen, the Netherlands; 2 Rheumatology, Sint Maartenskliniek 6574 NA Ubbergen, the Netherlands; 3 Rheumatology, Radboud University Medical Center, 6525 GA Nijmegen, the Netherlands; 4 Frazer Institute, The University of Queensland, Brisbane, QLD 4102, Australia; 5 Institute for Chemical Immunology, Radboud University Medical Center, 6525 GA Nijmegen, the Netherlands; 6 Department of Biochemistry and Pharmacology, Bio21 Molecular Science and Biotechnology Institute, The University of Melbourne, Parkville, VIC 3052, Australia

**Keywords:** Immunology, Immune response, Components of the immune system

## Abstract

Inflammatory arthritis (IA) is characterized by persistent joint inflammation and immune cell infiltration, including CD1c^+^ dendritic cells (DCs), comprising DC2s and DC3s. To investigate their developmental and functional specialization in IA, we characterized DC2s and DC3s in the peripheral blood (PB) and synovial fluid (SF) of patients with IA. DC3 frequencies were increased in PB of patients with juvenile idiopathic arthritis and correlated with disease activity in early rheumatoid arthritis. While PB DC3s showed strongly impaired T cell activation, SF DC3s induced only marginally lower T cell proliferation compared to DC2s and primed higher frequencies of IL-17^+^ and IFNγ^+^ T cells. Furthermore, SF from patients with IA induced the DC3 phenotype in DC2s from healthy donors, an effect abrogated by IL-6 receptor blockade and dependent on JAK/STAT3 signaling. Altogether, these findings reveal the impact of tocilizumab and JAK inhibitors on inflammatory DC3s in IA and offer mechanistic insights for IA treatment.

## Highlights

• DC3s frequency increases in juvenile patients with arthritis
• Synovial fluid DC3s induce more IL-17^+^ CD4 T cells and IFNγ^+^ CD4 T cells than DC2
• DC3s in arthritis can arise from DC2, not solely from an independent progenitor
• Synovial fluid derived IL-6 drives DC2 to DC3 conversion via JAK/STAT3 signaling

## Graphical abstract

**Figure:**
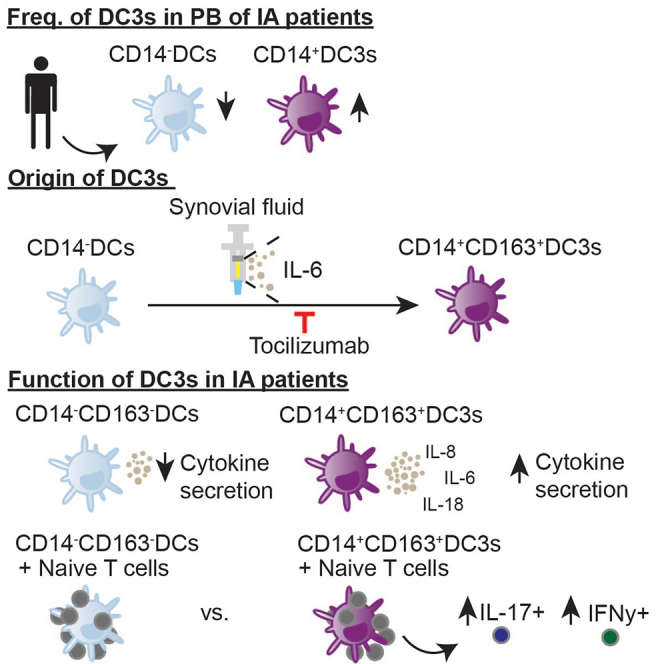

## Introduction

Inflammatory arthritides (IA) are a diverse group of conditions characterized by persistent joint inflammation, leading to pain and progressive joint destruction.^1^ In adults, the two most common diseases within IA are rheumatoid arthritis (RA) and psoriatic arthritis (PsA).^2^^,^^3^ In children, juvenile idiopathic arthritis (JIA) is the major pediatric condition of IA.^4^^,^^5^ In patients with IA both the adaptive and innate immune systems are involved. While the contributions of autoreactive B cells, CD4 T cells, and cytotoxic CD8 T cells to altered immune responses have been well characterized,^6^ the role of dendritic cells (DCs) is less understood.^6^

DCs are found in peripheral tissues and lymphoid organs where they play critical roles in innate and adaptive immunity.^7^^,^^8^ The human DC family is divided into subsets based on their distinct ontogeny, phenotype, transcriptome, and function.^9^^,^^10^ Plasmacytoid dendritic cells (pDCs) specialize in the production of type I interferon, which is important for viral defense. They are also involved in autoimmune disorders such as systemic lupus erythematosus and psoriasis.^11^^,^^12^^,^^13^ CD141^+^ type 1 conventional DCs (cDC1), and CD1c^+^ type 2 conventional DCs (DC2) subsets are capable of inducing naive T cell proliferation and shaping diverse T cell responses.^14^ cDC1s are known to efficiently present antigens to cytotoxic CD8 T cells, whereas DC2s are superior in polarizing T-helper (Th)1, Th2, and regulatory T cell responses.^14^^,^^15^^,^^16^ Type 3 DCs (DC3s) were only recently discovered in the blood of healthy individuals following single-cell RNA-seq analysis.^17^

DC3s are defined by the expression of CD1c as well as the monocyte/macrophage markers CD14 and CD163. Although progress has been made in understanding the ontogeny of DC3s, their development warrants further characterization.^18^^,^^19^ DC3s have been obtained via DC2-independent developmental pathways, either from CD34^+^ hematopoietic stem and progenitor cells (HSPCs) driven by granulocyte-macrophage colony-stimulating factor^20^ or from IRF8^low^ granulocyte-monocyte-DC progenitors (GMDP).^21^ In contrast, Dutertre et al.^19^ hypothesizes that DC3s could develop from DC2s along a continuous spectrum, ranging from CD5^+^DC2s to CD5^−^CD163^−^ to CD163^+^CD14^−^, and subsequently to CD163^+^CD14^+^ DC3s.^19^ A developmental trajectory shared with DC2s is furthermore reported in cancer, where interleukin 6 (IL-6), Prostaglandin E2 (PGE2) and macrophage colony stimulating factor (M-CSF) secreted by cancer cells converted DC2s to DC3s.^18^^,^^22^ Functionally, steady-state DC3s have been shown to secrete more pro-inflammatory cytokines than DC2s, such as IL-6 and IL-1β. They have furthermore been reported to steer Th1- and Th17-type responses, though the full repertoire of their functional properties is still being established.^14^^,^^18^^,^^19^^,^^23^

In autoimmunity, DCs present (auto-) antigens and prime T cells, leading to the proliferation of T cells and antibody producing B cells, which contributes to the initiation and propagation of autoimmune responses.^24^ Data from several studies suggest that DCs in IA promote joint inflammation, that the deregulation of DCs can lead to autoimmunity, and that specific DC subsets are linked to treatment resistance.^25^^,^^26^ In patients with IA, few studies comparing DC2s and DC3s have been performed. These studies show that CD1c^+^DCs (comprising both DC2s and DC3s) are decreased in peripheral blood (PB) and increased in the SF (SF) of affected joints.^27^^,^^28^^,^^29^ In the SF, CD1c^+^DCs present with an activated and mature phenotype, enabling them to activate and polarize naive T cells toward various T cell subsets.^6^^,^^27^^,^^28^^,^^29^ These include IL17^+^ CD4 T cells, which are implicated in IA.^6^^,^^27^^,^^30^^,^^31^ After entering the synovial tissue, CD1c^+^DCs produce inflammatory mediators that further support the perpetuation of IA.^27^^,^^32^ Interestingly, DC3s specifically have been reported to infiltrate the synovium in patients with osteoarthritis (OA), a type of arthritis that is considered non-inflammatory, although evidence that low-grade inflammation is involved in its pathogenesis is increasing.^33^ In SF from patients with IA, an inflammatory DC subset expressing CD1c and the DC3-marker CD14 was found. They appeared to be a potent stimulator of Th17 cells, suggesting these represent DC3s.^32^ These findings indicate the involvement of DCs in arthritis pathogenesis, yet underline the limited number of studies subdividing the heterogeneous CD1c^+^ DC population into DC2s and DC3s. This precludes a complete understanding of subset-specific characteristics.^6^^,^^28^ Because recent studies have shown that DC3s are increased in several immune-mediated diseases^18^^,^^19^^,^^34^ it is important to establish how DC3s develop in IA and in which functional capacity. Investigating the functional abilities of DC2s and DC3s in PB and SF will advance our understanding of the subset-specific contribution of DC2 and DC3 to IA pathogenesis.

In this work, we set out to characterize DC2 and DC3 subsets in the inflammatory environment of arthritis. Insights into the development of DC3s in IA, including the effects of the local inflammatory environment within the joint, will assist in understanding their contribution to the pathophysiology of inflammatory and autoimmune diseases and pave the way for potential therapeutic targeting. We hypothesize that the inflammatory environment of IA enhances DC3 development and expands DC3 numbers, potentially from DC2s as precursors. Additionally, investigating the functional abilities of DC2s and DC3s residing in PB and SF will be critical for our understanding of the subset-specific contribution of DC2 and DC3 to the disease pathogenesis. We found increased CD14^+^ DC3s in the PB of JIA. In early RA (ERA) CD14^+^ DC3s frequencies correlated with disease activity. By assessing CD14^+^ DC3s circulating in the PB of patients with arthritis and those present in the SF, the local site of inflammation, we delineated how the microenvironment can affect DC3s. Whereas CD14^+^DC3s in PB exhibit minimal T cell activating capacity, those isolated from SF exert a striking pro-inflammatory phenotype with potent T cell activation capacity, directing T cells toward Th17 cells. In addition, DC3s showed increased IL-6 and IL-8 secretion. Utilizing SF from patients with arthritis, we show that DC3s can develop from DC2s in response to IL-6 and JAK/STAT3 signaling. Overall, our study provides important insights into DC3 development in arthritis and emphasises the importance of examining the individual CD1c^+^ DC subsets to understand their relative contribution to pathogenic processes.

## Results

### Increased DC3s with impaired T cell activation in recent onset arthritis and juvenile idiopathic arthritis

**Figure 1:**
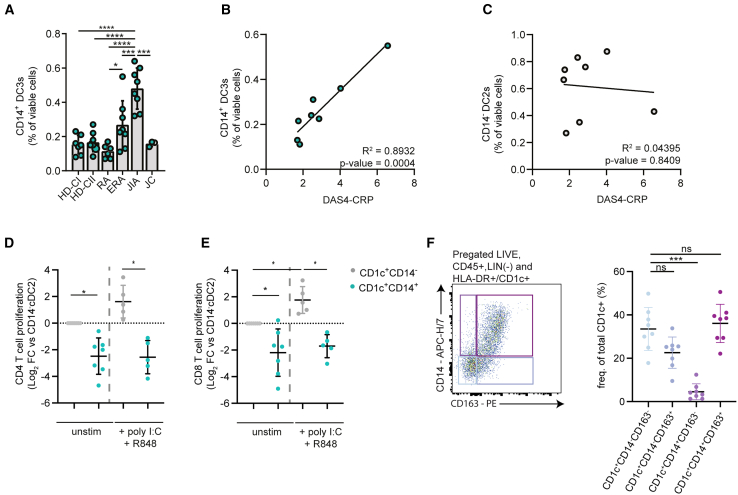
Peripheral blood of patients with active recent-onset rheumatoid arthritis and juvenile idiopathic arthritis comprises increased frequencies of CD14^+^ DC3s with strongly impaired T cell activation (A) Frequency of CD14^+^ DC3s of total viable peripheral blood mononuclear cells (PBMCs) determined by flow cytometry for a cohort of healthy donors from Translational Research Institute Brisbane (HD-CI) and Radboudumc Nijmegen (HD-CII), a cohort of patients with long-standing rheumatoid arthritis (RA), early RA (ERA), and juvenile idiopathic arthritis (JIA), with juvenile healthy donors as control cohort (JC). Each symbol represents an individual donor (mean ± SD; one-way ANOVA and Tukey’s multiple comparisons test). (B–E) Disease activity score (DAS4-CRP) of patients with ERA correlated with the frequencies of CD14^+^ DC3s and in (C) with the frequencies of CD14^−^ DC2s in PBMCs. Correlation was statistically tested with Pearson’s correlation coefficient and two-tailed *p* value. Allogeneic CD4 (D) and CD8 (E) T cell proliferation after a 5-day co-culture with immature and poly I:C + R848-matured CD14^+^ and CD14^−^ DC2s, isolated and sorted from the peripheral blood of patients with arthritis. Each symbol represents an individual donor, and frequencies are normalized to unstimulated CD14^−^ DC2s (mean ± SD; mixed-effects analysis with Fisher’s LSD). (F) Frequency of CD1c^+^DC subpopulations from HLA-DR^+^CD1c PBMCs in patients with IA. Representative flow cytometry gating for CD14 and CD163 expression is shown on the left for an IA patient. Each symbol represents an individual donor (*n* = 8, mean ± SD; one-way ANOVA followed by Tukey’s test). ∗*p* < 0.05, ∗∗*p* < 0.01, ∗∗∗*p* < 0.001, ∗∗∗∗*p* < 0.0001. See also Figures S1A–S1C.

To evaluate CD14^+^ DC3s in arthritis, we first compared their frequencies in the PB of patients with IA and healthy donors (HDs) using flow cytometry (Figure S1A). We included peripheral blood mononuclear cells (PBMCs) from adults with long-standing and recent-onset RA (ERA), and juvenile idiopathic arthritis (JIA). While patients with long-standing RA do not show increased frequencies of CD14^+^ DC3s compared to HDs, patients with ERA had almost twice the amount of CD14^+^ DC3s compared to HDs (0.27% vs. 0.15% of PBMCs) and significantly increased frequencies compared to long-standing RA (0.11% of PMBCs) (Figure 1A). Notably, all patients with long-standing RA were receiving treatment, whereas patients with ERA were either untreated or had only recently started treatment. Interestingly, the frequency of CD14^+^ DC3s in patients with JIA was significantly higher than in any other group, with CD14^+^ DC3s on average forming 0.48% of the PBMCs. The frequency of CD14^+^ DC3s in age-matched juvenile HDs was comparable to that of adult HDs (0.16% of PBMCs) and significantly lower than the JIA cohort (Figure 1A). The CD14^+^ DC3 frequency was correlated with disease activity in the ERA cohort, as assessed by the 4-variable DAS4-CRP,^35^ underlining the clinical relevance of CD14^+^ DC3s in arthritis^35^ (R^2^ 0.8932, *p* = 0.0004) (Figure 1B). In contrast, no correlation was found between CD14^−^DC2s and DAS4-CRP score (R^2^ 0.04395, *p* = 0.8409) (Figure 1C). To obtain insights into the functional capabilities of CD14^−^DC2s and CD14^+^ DC3s in the PB of patients with arthritis, we assessed the ability of sort-purified DC2s and DC3s to induce the proliferation of allogeneic T cells derived from HDs (Figure S1B). In those patients, CD14^−^DC2s induced a geometric mean (GM) of 16.6% proliferating CD4 T cells. In contrast, CD14^+^ DC3s induced 5.5 times lower levels of CD4 T cell proliferation (GM 3%, log_2_ FC -2.48) (Figure 1D). For CD8 T cells, proliferation induced by CD14^+^ DC3s was lower than that induced by CD14^−^DC2s by a log_2_ fold change of −2.2 (Figure 1E). In addition, while T cell activation was enhanced when CD14^−^DC2s were matured with poly I:C and R848, no increase in T cell proliferation occurred after stimulation with matured CD14^+^ DC3s (Figures 1D and 1E). These data suggest that in PB of patients with IA, DC3 are inferior to DC2s in their capability to activate T cells.

In recent years, in addition to CD14, the expression of CD163 has become important to delineate DC2s and DC3s.^9^^,^^19^ While in Figure 1A we compared the frequencies of CD14^+^DC3s from patients with IA to HDs, we now set out to investigate the relative abundance of DC2s and DC3s with both CD14 and CD163 at our disposal. For this characterization, we used PB of patients with IA from another cohort (including RA, PsA, and spondylarthritis. Using these surface markers, four subsets of CD1c^+^ DCs differentially expressing the cell surface markers CD14 and CD163 were identified: CD1c^+^CD14^−^CD163^−^ DC2s, the intermediate subsets CD1c^+^CD14^+^CD163^−^, CD1c^+^CD14^−^CD163^+^, and CD1c^+^CD14^+^CD163^+^ DC3s (Figures 1F and S1C). Notably, CD1c^+^CD14^+^CD163^-^ DCs are present at a very low frequency of 4.6% and a majority of CD1c^+^ DCs expressed CD163 and CD14 (Figure 1F). Furthermore, there is a population of CD14^−^DC2s with CD163 expression, which would be categorized as DC2s and not DC3s in Figure 1A. Taken together, we provide a first enumeration of CD14^+^ DC3s in IA and find increased frequencies in PB of patients with active arthritis, where they display a low immune-activating capacity. In addition, we observe that most CD1c^+^ DCs in the blood of patients with IA are CD163^+^, with CD14^+^DCs also being CD163^+^.

### Synovial fluid DC3s are proinflammatory

**Figure 2:**
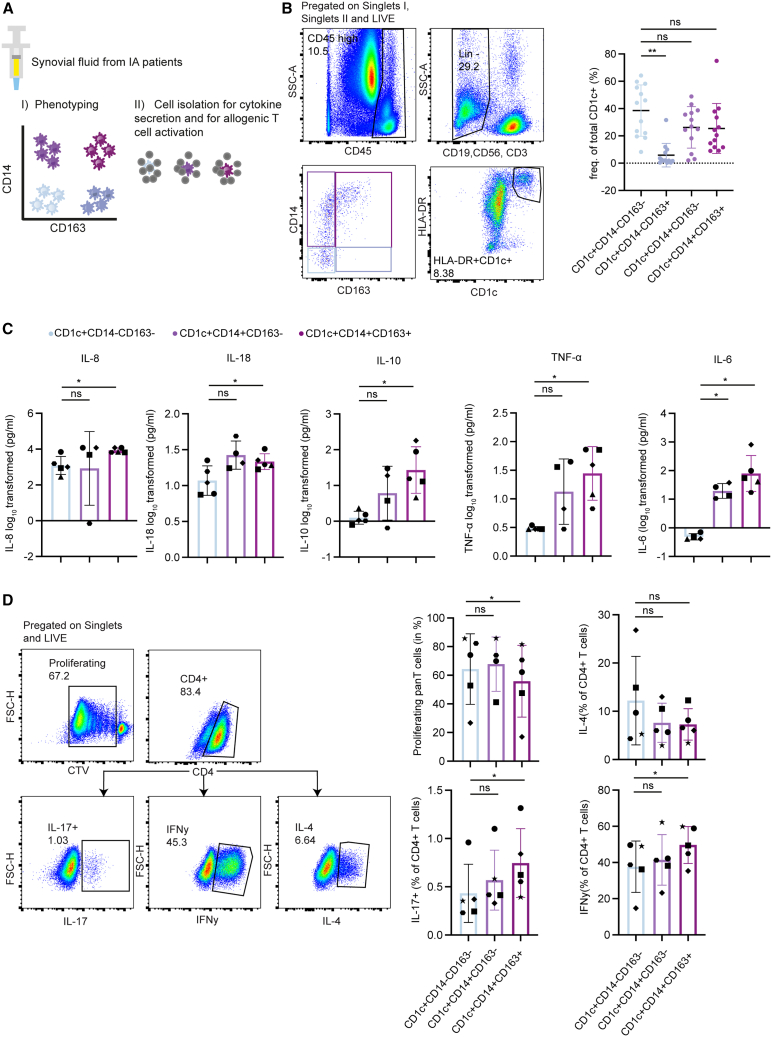
CD1c^+^CD14^+^CD163^+^ DC3s in SF have a pro-inflammatory phenotype (A) Schematics of the assays used in panels B–D (B) Representative gating strategy for flow cytometry analysis of CD1c^+^ DC subsets in SF of patients with IA used for the enumeration (B, right). Each dot represents one patient (mean ± SD, *n* = 13, one-way-ANOVA followed by Tukey’s test). (C) CD1c^+^ DCs were sorted from SF of patients with IA into the three depicted subsets based on CD163 and CD14 expression, cultured for 21 h and cytokine levels measured by the LEGENDplex Human inflammation panel (mean ± SD, *n* = 5 patients, each the mean of technical replicates). Data were log_10_-transformed, and significance tested by one-way ANOVA followed by Tukey’s test. (D) CD1c^+^ DCs from SF of patients with IA were FACS sorted into three subsets based on CD14 and CD163 expression, rested for 21 h and subsequently co-cultured for 6 days with CTV/CSFE labeled naive allogenic T cells. Representative gating strategy for T cell subsets is shown on the left. Plots of frequencies of IFNγ^+^(Th1), IL-4^+^ (Th2) or IL-17^+^ (Th17) producing CD4^+^ T cells as a percentage of proliferating CD4 T cells. Each connecting line represents one patient (mean ± SD, *n* = 5, each the mean of technical replicates). Donor depicted with a Rhombus was cultured for 10 days instead of 6, and proliferating cells were gated based on SSC-A and FSC-A instead of CTV staining. Significance tested by one-way-ANOVA followed by Tukey’s test. ∗*p* < 0.05, ∗∗*p* < 0.01, ∗∗∗*p* < 0.001, ∗∗∗∗*p* < 0.0001. See also Figures S2A–S2E.

IA is characterized by inflamed joints that contain an excessive amount of SF infiltrated by immune cells. We examined the features of CD1c^+^ DC subsets in SF of patients with IA (Figure 2A).

Using flow cytometry, we analyzed the four subsets of CD1c^+^ DCs differentially expressing CD14 and CD163 on their surface (Figures 2B and S2A). We observed the lowest frequencies for CD1c^+^CD14^−^CD163^+^ DCs in SF (Figure 2B), in contrast to our results in PB (Figure 1F). In PB we observed CD1c^+^CD14^+^CD163^-^ DCs to be the least abundant subset. The major histocompatibility complex (MHC) class II receptor HLA-DR and DC maturation marker CD86 were highly expressed on all subsets, and no statistically significant differences were observed between the subsets (Figure S2C).

As the secretion of pro-inflammatory cytokines plays a pivotal role in IA, we sought to determine the cytokines produced by the different DC subsets in SF.^36^^,^^37^ Sorted DC subsets (Figure S2B) were cultured for 21 h, followed by assessing cytokine production using the LEGENDplex Human inflammation panel. All SF DCs produced detectable levels of IL-33, IL-1β IL-23, IL-18, IL-8, and CCL2 (Figures 2C and S2D). For IL-12, IL-17, IFNα2, and IFNγ the levels were under the detection limit for most donors (Table S6). The cytokines IL-8, IL-18, IL-10, TNF-α, and IL-6, were produced in higher amounts by CD1c^+^CD14^+^CD163^+^ DC3s than CD1c^+^CD14^−^CD163^−^DC2s. Notably, TNF-α, IL-10 and IL-6 were hardly detectable in CD1c^+^CD14^−^CD163^−^DC2s (Figure 2C). IL-10, TNF-α, and IL-6 showed a gradual increase from CD1c^+^CD14^−^CD163^−^DC2s to CD14^+^CD163^−^ DC3s to CD1c^+^CD14^+^CD163^+^ DC3s. Albeit not significant, a trend toward higher secretion of CCL-2 and IL-1β by CD1c^+^CD14^+^CD163^+^ DC3s was observed. IL-23 and IL-33 were produced in similar amounts across the SF DC subsets (Figure S2D).

Next to the cytokine secretion profile, we assessed the naive T cell polarization capacity of CD1c^+^CD14^−^CD163^−^DC2s, CD1c^+^CD14^+^CD163^−^DC3s, and CD1c^+^CD14^+^CD163^+^DC3, while omitting the CD1c^+^CD14^−^CD163^+^ subset due to its low frequency (Figures 2B and S2B). CD1c^+^CD14^+^CD163^+^DC3s induced slightly lower proliferation of allogeneic pan T cells compared to DC2, which is similar to PB DC3s but less pronounced (Figure 2D). Expression levels of HLA-DR and CD86 were comparable between the different DC subsets (Figure S2C). Regarding the priming of the specific CD4 T cell subsets, CD1c^+^CD14^+^CD163^+^ DC3s in SF showed a trend toward reduced amounts of IL-4^+^ CD4 T cells and a significantly higher proportion of IFNγ^+^ CD4 T cells (Figure 2D). Moreover, we observed that a significantly higher percentage of IL-17^+^ CD4 T cells was induced by CD1c^+^CD14^+^CD163^+^ DC3s compared to CD1c^+^CD14^−^CD163^-^ DC2s (Figure 2D). Taken together, CD1c^+^CD14^+^CD163^+^ DC3s are pro-inflammatory with the capacity to skew the differentiation of CD4 T cells toward IFNγ^+^ and IL-17^+^ CD4 T cells.

### SF from patients with arthritis induces DC3-like cells *in vitro*

**Figure 3:**
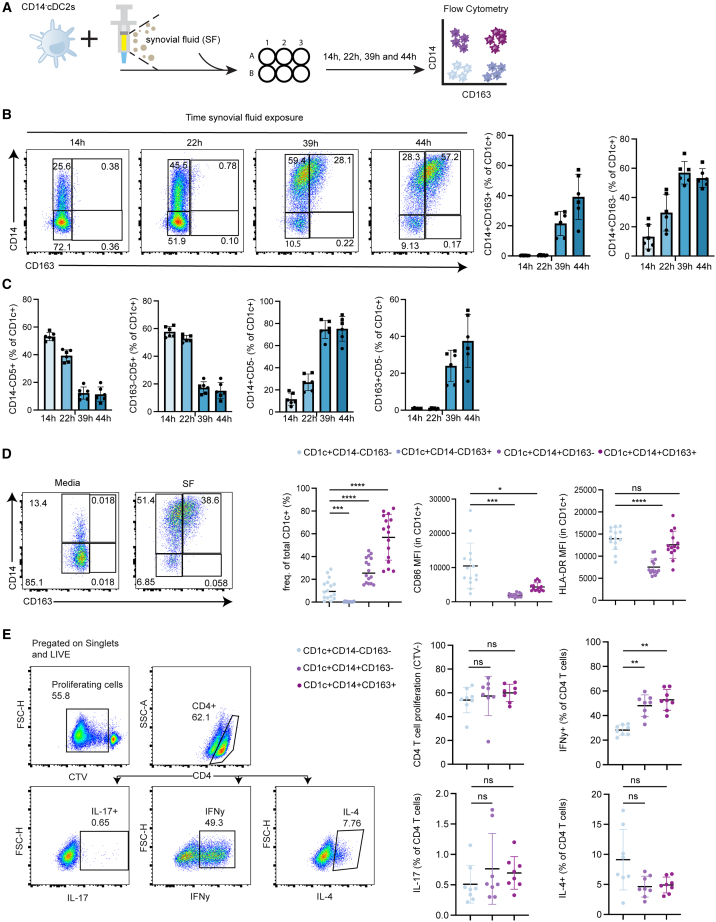
SF from patients with arthritis induces CD1c^+^CD14^+^CD163^+^ DC3s with similar T cell polarizing capacities as *in vivo* DCs (A) Schematics for assay B-C. (B) Frequencies of CD1c^+^CD14^+^C163^+^ DC3s and CD1c^+^CD14^+^C163^-^ DC3s after the isolation of CD14^−^DC2s from blood of healthy donors (*n* = 2) depicted with square and dot and cultured with 50% hyaluronidase-treated SF from patients with arthritis (*n* = 3) for 14 h, 21 h, 39 h and 44 h (mean ± SD, *n* = 3). Representative flow cytometry gating for CD14 and CD163 expression over time is shown on the left. (C and D) Frequencies of CD1c^+^CD14^−^CD5^+^ DCs, CD1c^+^CD163^−^CD5^+^ DCs CD1c^+^CD14^+^CD5^−^ DCs and CD1c^+^CD163^+^CD5^−^ DCs, after the isolation of CD14^−^DC2s from blood of healthy donors (*n* = 2) depicted with square and dot and cultured with 50% hyaluronidase-treated SF from patients with arthritis (*n* = 3) for 14 h, 21 h, 39 h and 44 h (mean ± SD, *n* = 3) (D) Enumeration and phenotype of subsets used in (B–C) after co-culture with 50% hyaluronidase-treated SF from patients with arthritis (*n* = 18) for 2 days including representative flow cytometry plots. Each dot represents one SF donor (mean ± SD, *n* = 18). MFI; median fluorescence intensity. (E) Naive T cell activation by sorted DC-subsets from (D) after 6-day co-culture. Representative gating strategy is shown on the left. Plots represent frequencies of IFNγ^+^(Th1), IL-4^+^ (Th2) or IL-17^+^ (Th17-) producing CD4^+^ T cells as a percentage of proliferating CD4 T cells. Each dot represents one SF donor (mean ± SD, *n* = 8, each the mean of technical replicates) Significance tested by one-way-ANOVA followed by Tukey’s test. ∗*p* < 0.05, ∗∗*p* < 0.01, ∗∗∗*p* < 0.001, ∗∗∗∗*p* < 0.0001. See also Figures S3A–S3C.

The frequencies of CD1c^+^CD14^+^ DC3s in PB of patients with RA correlating with disease activity, combined with the high levels of pro-inflammatory CD14^+^ DC3s in SF, suggests that proinflammatory cytokines driving IA may also drive the development of CD14^+^ DC3s. To understand the effects of the cytokine-rich environment of the joint,^38^ we sought to investigate the effect of SF on DC3 development. In addition, we looked at the ability of the myeloid cells DC2s and monocytes to differentiate into DC3s in response to SF. To address the first question, we isolated CD14^−^DC2s from PB of HDs and started *in vitro* differentiation assays with SF from patients with IA, to mimic arthritis cues, over several time periods (Figure 3A). We observed that CD14^−^DC2s started to express CD14 after 14 h but lacked CD163 expression (Figures 3B and S3B). After 39 h of incubation with SF, CD163 was expressed and on average 22% of the cells were CD1c^+^CD14^+^CD163^+^ DC3s, which further increased to 39% at 44 h (Figure 3B). Furthermore indicative for a phenotypic shift was that at 14 h DC2s were mostly positive for CD5, a marker associated to be absent in DC3s,^19^ and negative for CD163 (Figures 3C and S3B). At 39 h there was a drop in CD5 expression (Figure S3B), with a concomitant increase in CD14 and CD163 (Figure 3C), with most cells being positive for CD14 and CD163 but negative for CD5 (Figure 3C, Figure S3B). Culturing in media did not induce CD14^+^CD163^+^ DC3s (Figure S3C). This indicates that in the presence of SF, a gradual phenotypic change takes place from CD14^−^ DC2s to CD14^+^CD163^-^ DCs and subsequently to CD14^+^CD163^+^ DCs. Notably, alongside DC2s, we examined monocytes as precursors for inflammatory DC3s. Co-cultures with SF for two days failed to induce notable CD1c expression in monocytes (Figure S3D). In conclusion, we show that DC3s can be derived from PB DC2s and not monocytes in the presence of SF from patients with IA.

To obtain sufficient amounts of CD163^−^ and CD163^+^ DCs from CD14^−^DC2s of HDs, a two-day culture period was selected for future experiments. CD14^+^CD163^+^ DC3 induction from DC2s by SF of 18 different patients with IA ranged from 26% to 82% (Figure 3D). *In vitro* induced DC3s showed a significant reduction in CD86 and similar expression of HLA-DR (Figure 3D). Next, we examined their naive T cell polarization capacity to determine whether this would be comparable to DC3s *ex vivo*. After a two-day culture period with SF, DCs were sorted into CD14^−^CD163^-^, CD14^+^CD163^-^, and CD14^+^CD163^+^ DC3s. CD14^−^CD163^+^ DC frequencies were too low for functional assays (Figure 3D). Sort-purified DCs were co-cultured with naive allogenic T cells for six days, after which proliferation and polarization were assessed. There was no difference in CD4 T cell proliferation induced by the different DC subsets (Figure 3E). There was a significant increase in IFNγ-producing T cells after co-culture with CD14^+^CD163^+^ DC3s compared to CD14^−^CD163^−^ DC2s (Figure 3E) and this effect was similar to the effect of DC3s *ex vivo*. Further in line with the SF DCs *ex vivo* is the decreasing trend of IL-4^+^ CD4 T cells, albeit not significant in both cases. The increase in IL-17^+^ CD4 cells seen for SF CD14^+^CD163^+^ DC3s was not detected here (Figure 3E). Taken together, these data suggest that DC2s can be converted into DC3s by SF with the capacity to polarize naive T cells toward Th1, comparable to their *in vivo* SF counterparts.

### interleukin-6 in SF induces the differentiation of DC2s into DC3s

**Figure 4:**
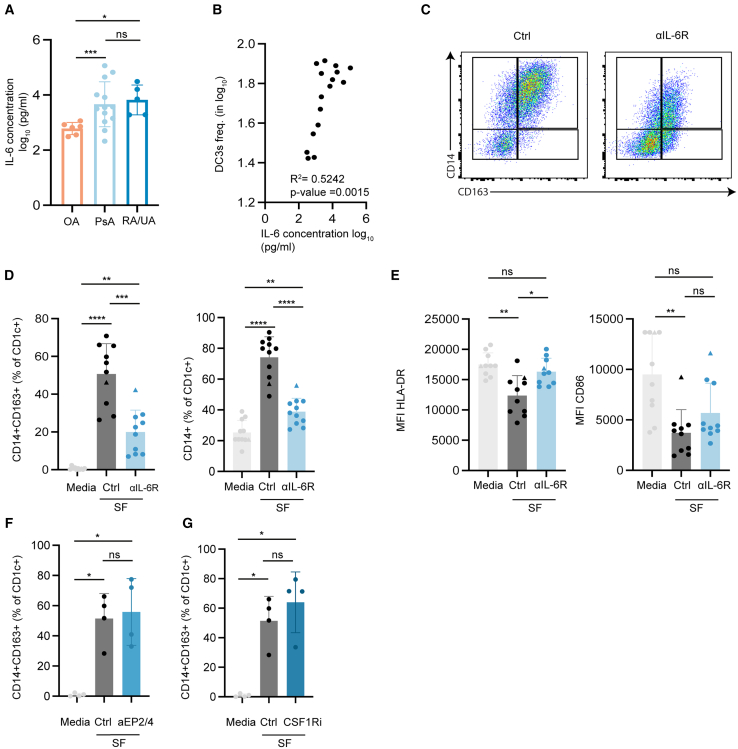
Modulation of DC2s with anti-IL-6R prevents SF induced DC3s (A) IL-6 ELISA on Hyaluronidase-treated SF from patients with osteoarthritis (OA, *n* = 6), psoriatic arthritis (PsA, *n* = 10), and rheumatoid arthritis (RA/UA *n* = 6) (mean ± SD). Values were log_10_ transformed and plotted on a linear scale (one-way-ANOVA followed by Tukey’s test). (B) Correlation between IL-6 concentration in SF as determined by IL-6 ELISA (*n* = 16 patients) with the frequency of CD1c^+^CD14^+^CD163^+^D3Cs after a two-day culture period, starting with CD14^−^ DC2s. Frequency and IL-6 concentration were log_10_ transformed and tested for correlation using Pearson’s correlation coefficient and two-tailed *p* value. (C–E) CD14^−^DC2s were isolated from the blood of healthy donors and cultured with media or 50% hyaluronidase-treated SF from patients with arthritis. The frequency (D) and phenotype (E) were measured in the presence or absence of 10 μg/mL tocilizumab (αIL-6R) or isotype control antibody. Triangle indicating a non-responsive patient. Representative flow cytometry plots are shown in (C). (mean ± SD, *n* = 10), MFI; median fluorescence intensity. (F–G) CD14^−^DC2s were isolated from the blood of healthy donors and cultured with media or 50% hyaluronidase-treated SF from patients with arthritis. The frequency was measured in the presence or absence of (F) soluble aEP2/4 (30μM of aEP2 and 5μM of aEP4) or (G) 100nM sunitinib or DMSO control. (mean ± SD, *n* = 4), Significance tested by one-way-ANOVA followed with Tukey’s test. ∗*p* < 0.05, ∗∗*p* < 0.01, ∗∗∗*p* < 0.001, and ∗∗∗∗*p* < 0.0001. See also Figure S4A.

Cytokines are main effectors in the pathogenesis of IA, implicated in promoting autoimmunity, chronic inflammation, and tissue destruction.^39^ To determine which soluble mediators are involved in the conversion of DC2s to DC3s by SF we focused on IL-6, considering its importance in the pathology of IA, the therapeutic potential of IL-6 receptor blockers,^40^^,^^41^ and its known ability to facilitate the differentiation of CD14^−^DC2s to DC3s in cancer.^18^ We detected varying concentrations of IL-6 in the SF of patients with IA depending on the type of arthritis (Figures 4A and S4A). SF from patients with OA contained an average of 671 pg/mL, whereas SF from patients with PsA and RA contained an average of 20158 pg/mL and 11550 pg/mL, respectively (Figure 4A). Arthritis patients with IA and OA were included in the subsequent correlation analysis between IL-6 and DC3 frequencies, to cover a broad range of IL-6 levels. Interestingly, the IL-6 concentration in SF and the frequency of DC3s induced by the corresponding SF were significantly correlated (Figure 4B). To assess the contribution of IL-6 to DC3 development, we investigated whether blocking IL-6 signaling with tocilizumab, a humanized monoclonal antibody that binds the interleukin-6 receptor (IL-6R), would prevent the formation of DC3s by SF. Culturing CD14^−^DC2s in the presence of SF from patients with IA with tocilizumab decreased CD1c^+^CD14^+^CD163^+^ DC3s from 50% with SF alone to 20% in the presence of SF and anti-IL-6R (Figures 4C and 4D). Furthermore, total CD1c^+^CD14^+^ DC frequencies decreased from 76% to 37% in the presence of SF and anti-IL-6R (Figure 4D). The reduced expression of HLA-DR in total CD1c^+^ DCs was rescued by the addition of tocilizumab, which was mainly facilitated by the reduced number of DC3s that have lower HLA-DR (Figure 4E). This was not the case for CD86 (Figure 4E). Only one SF donor (Patient 2, Figure S3E) was not affected by the tocilizumab treatment (Figure 4D, triangles). This patient suffered from reactive arthritis, high CRP, and exceptionally high IL-6 levels around 400 ng/mL*.* These levels are possibly too elevated to sufficiently inhibit signaling. In addition to IL-6, IL-1β, and TNF-α are key pro-inflammatory cytokines detected in SF of patients with IA, where they play important roles.^42^^,^^43^^,^^44^ Therefore, we assessed their ability to induce a CD14^+^DC3 phenotype. However, neither recombinant TNFα nor IL-1β, alone or in combination, promoted DC3 induction. Instead, they appeared to inhibit CD14 expression when compared to the media control (Figure S4B).

Several studies have reported that in addition to IL-6, tumor-derived M-CSF and PGE2 are capable of inducing DC3 development.^18^^,^^45^ We investigated if M-CSF and PGE2 contribute to SF-induced DC3 development. Sunitinib, a small-molecule receptor tyrosine kinase inhibitor,^46^ was used to block M-CSF signaling, while PGE2 activity was inhibited using two antagonists targeting the Prostaglandin E_2_ receptor 2 (EP2) and EP4.^47^ However, neither M-CSF nor PGE2 inhibition reduced CD1c^+^CD14^+^CD163^+^ DC3s frequencies in SF (Figures 4F and 4G). In summary, IL-6 plays a key role in the conversion of DC2 to DC3s, whereas TNFα, IL-1β, M-CSF, and PGE2 do not contribute to DC3 development in the IA microenvironment.

### JAK/STAT3 signaling downstream of the interleukin-6 receptor is important for the conversion of DC2s into DC3s

**Figure 5:**
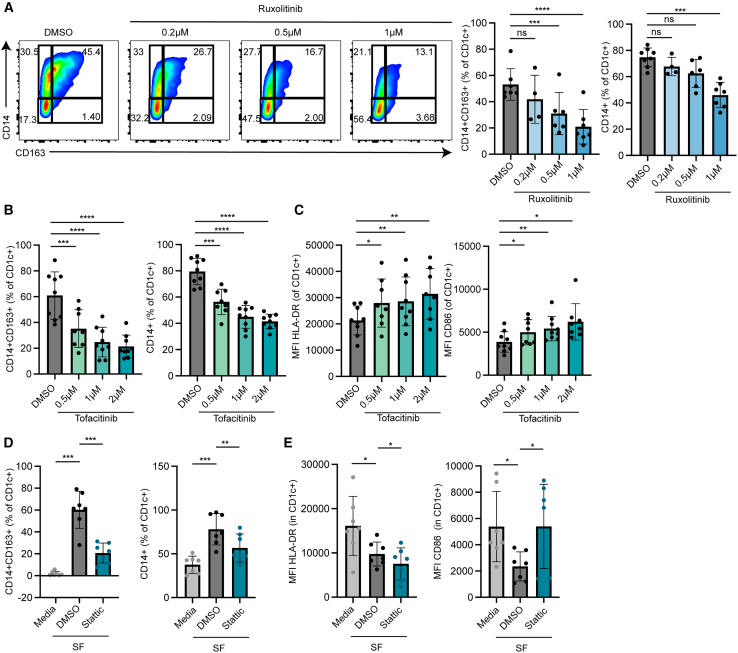
Modulation of DC2s with JAK/STAT3 small molecule inhibitors prevents synovial induced DC3s (A–E) CD14^−^ DC2s were isolated from blood of healthy donors and cultured with 50% hyaluronidase-treated SF from patients with arthritis except for the media conditions in (D+E), in the presence or absence of indicated concentrations of JAK inhibitors ruxolitinib (A) (*n* = 5 DCs, *n* = 5 SF) and (B+C) tofacitinib (*n* = 6 DCs, *n* = 7 SF) or (D-E) STAT3 inhibitor static (1 μM). Each dot represents a biological replicate (*n* = 7, mean ± SD). MFI; median fluorescence intensity. Significance was tested versus DMSO control with a one-way ANOVA, Dunnet’s (A–C) or Tukey’s (D-E) multiple comparison test∗*p* < 0.05, ∗∗*p* < 0.01, ∗∗∗*p* < 0.001, ∗∗∗∗*p* < 0.0001. See also Figure S4C.

Having demonstrated that IL-6 is important for the conversion of DC2s to DC3s, we continued our investigation into downstream pathways potentially mediating this. One of the commonly known pathways through which IL-6 signals is the JAK/STAT3 pathway. JAK/STAT signaling can be efficiently blocked by several small-molecule inhibitors such as the pan JAK inhibitors ruxolitinib and tofacitinib, with the latter being used for the treatment of patients with arthritis.^48^^,^^49^ To investigate the role of JAK signaling in the induction of DC3s we treated CD14^−^ DC2s with various concentrations of ruxolitinib (JAK1/2 inhibitor) during a two-day culture with SF from patients with IA (Figure 5A). CD1c^+^CD14^+^CD163^+^ DC3 induction was compromised in a dose-dependent manner, suggesting JAK signaling is required for the conversion into DC3s by SF (Figure 5A). In addition, the frequency of CD1c^+^CD14^+^ DCs also decreased (Figure 5A). Ruxolitinib treatment during culture with SF did not influence total HLA-DR and CD86 expression (Figure S4C). Similar results were obtained with tofacitinib (JAK1/3 inhibitor), as only 35% of CD1c^+^ DCs became CD14^+^CD163^+^DC3s upon coculture with SF in the presence of the lowest concentration of tofacitinib, which further decreased with higher concentrations, as opposed to 60% when no drug was present (Figure 5B). Next to, that the frequency of total CD1c^+^CD14^+^ decreased and the total CD1c^+^ DCs cultured with SF and tofacitinib showed significantly increased expression of CD86 and HLA-DR (Figures 5B and 5C).

Finally, we investigated whether STAT3 downstream of JAK1/2 is important for the formation of DC3s. CD14^−^DC2s were treated with static, an inhibitor that binds to the SH2 domain of STAT3 and blocks IL-6 induced signaling.^50^ Similar to the JAK inhibitors, we observed a strong reduction in the frequency of CD1c^+^CD14^+^CD163^+^ DC3s, from 60% without drugs to 21% when cultured with stattic (Figure 5D). Moreover, the frequency of total CD1c^+^CD14^+^ DCs was reduced (Figure 5D). Similarly to tofacitinib, the expression of CD86 was increased to levels equal to those of the media control by static, whereas HLA-DR decreased (Figure 5E). Taken together, these results suggest that JAK/STAT3 is an important downstream signaling pathway of the IL-6R, contributing to the differentiation of DC3s.

## Discussion

Dendritic cells are pivotal in orchestrating immune responses and maintaining immune balance. In inflammatory arthritis this balance is disturbed, causing a systemic inflammatory response with inflamed synovial joints. Although CD1c^+^DCs play a crucial role in regulating immune balance, functional data on DC subsets from patients with IA are scarce. In this work, we characterized DC2s and DC3s in PB and SF of patients with IA. CD14^+^ DC3 frequencies were increased in patients with JIA. In addition, CD14^+^ DC3 frequency correlated strongly with disease activity in ERA. Functionally, CD14^+^CD163^+^ DC3s retrieved from the local inflammatory site were potent naive T cell stimulators, albeit less than CD14^−^CD163^-^ DC2s. In addition, they were superior in inducing IL-17^+^ CD4 T cells and IFNγ^+^ CD4 T cells, whereas PB DC3s were not. Finally, we demonstrated that CD14^+^CD163^+^ DC3s develop from DC2s, but not from monocytes, in response to IL-6 in the SF and mediated through JAK/STAT3 signaling.

Our enumeration of CD1c^+^ DC subsets in SF revealed that the majority of CD1c^+^ DCs consisted of CD14^−^CD163^−^DCs. The frequencies showed high variability across patients, which most likely depends on the arthritis subtypes, as our analysis included primarily patients with RA, JIA, and PsA, on different treatments. Interestingly, there is a difference in PB and SF DC3 subsets. We observed that CD14^−^CD163^+^DCs and CD14^+^CD163^+^ DCs are detected in PB, whereas in SF, we mostly see CD14^+^CD163^-^ and CD14^+^CD163^+^DCs. Some studies have looked at DC3s in PB and observed a similar distribution of CD14 and CD163 on CD1c^+^DCs.^19^^,^^34^ Cuenca-Escalona et al.^45^ looked at CD1c^+^ DCs after adding ascites fluid from patients with ovarian cancer and observed a distribution similar to what we see in SF with the up-regulation of CD14 and CD163.^45^ These results reveal a changing expression patterns and phenotypes depending on the environment being the circulation (PB) or a more local inflammatory environment (SF).

The presence of CD1c^+^CD14^+^CD163^+^ DC3s in SF from patients with arthritis aligns with the reports of CD163^+^ DC3s detected in SF and tissue in OA, and CD1c^+^CD14^+^ DCs described in inflammatory tumor ascites and RA SF.^32^ A study by Moret et al.^27^ reported only low amounts of CD14 expressing CD1c^+^ DCs in both PB and SF of patients with long-standing RA.^27^ Noteworthy, in our study CD1c^+^CD14^+^DC3s in PB were only increased in patients recent-onset RA (compared to long-standing RA) and in patient with JIA compared to healthy individuals, which differs from the patient population investigated by Moret et al.^27^ In agreement with Moret et al.,^27^ circulating CD14^−^ DC2s were more abundant than CD1c^+^CD14^+^ DC3s within individual patients. Since the aforementioned studies and our study were started, additional markers such as CD88 and FCε RI^19^ have been discovered to better define DC3s and exclude monocytes. In addition, at the time of our PB characterization, CD163 was not yet defined as a DC3 marker and was therefore not taken along in those experiments. The absence of CD88, FC ε RI, and in some cases CD163, in the work described here, is a limitation of this study. These markers should be used in future studies to enhance the accuracy of DC-subset identification. All data considered, differences in detected CD1c^+^CD14^+^ DC3s frequencies might be related to the type of arthritis, onset of disease, and disease activity. Hence, further studies using larger cohorts of patients with arthritis, preferably stratified by arthritis type and disease activity, are required to establish this.

We established that CD14^+^ DC3s in PB induce very low proliferation of CD4 and CD8 T cells in an allogeneic setup. Interestingly, TLR ligand stimulation improved the T cell activation capacity of CD14^−^ DC2s but failed to enhance the T cell proliferation induced by CD1c^+^CD14^+^ DC3s. Refractory to stimulation is typical of DCs in exhaustion or paralysis, suggesting CD1c^+^CD14^+^ DC3s in PB adapted to a state of tolerance or even paralysis.^51^^,^^52^ The low number of proliferating T cells hindered us to continue with additional naive T cell assays to study T cell subset polarization. Of note, because CD88, a monocyte-specific marker,^21^ was not used to confirm the purity of our DC subsets, the CD14^+^ DC3s fraction could have been contaminated with some monocytes. As monocytes are less effective in priming T cells, this may partly contribute to the observed reduction in T cell proliferation. In contrast to PB, CD14^+^CD163^-^ DC3s and CD14^+^CD163^+^ DC3s in SF of patients with IA both showed a pro-inflammatory phenotype compared to CD14^−^CD163^-^ DC2s in SF. In addition, they were potent cytokine producers with CD14^+^CD163^+^ DC3s producing IL-8, IL-18, IL-10, TNF-α, and IL-6 the most. Supplementary to these findings, several studies report a more mature and pro-inflammatory phenotype for CD1c^+^ DCs present in SF versus PB,^27^^,^^28^^,^^29^ and a more pro-inflammatory phenotype for DC3s.^19^^,^^53^ SF CD14^+^ DC3s are continuously exposed to a highly inflammatory environment, while blood CD14^+^ DC3s reside in the circulation.^54^ Considering the observed differences between PB DC3 and SF DC3s, it will be important to assess the exact pathogenic contribution of PB DC3s versus SF DC3s in subsequent studies. Moreover, pro-inflammatory SF DC3s might originate in PB and migrate to inflamed joints or differentiate locally, which remains to be investigated.

Further analysis of the capacity of SF DC subsets to polarize naive T cells showed that CD14^+^CD163^+^ DC3s have the strongest induction of Th17 cells, concordant with their high IL-6 production. In accordance with these findings, Segura et al.^32^ reported that CD14^+^ DCs from RA SF were superior Th17 inducers.^32^ Similarly, Iwabuchi et al.^23^ noticed that in humanized mice CD14^+^ DC2s primed and polarized naive CD4^+^ T cells toward Th1 cells more profoundly than CD14^−^ DC2s.^23^ Qiu et al.^33^ observed that both IFNγ and IL-17^+^ T cells increased after priming with CD163^+^ DCs compared to macrophages in OA.^33^ Further supporting the DC3-specific function of priming Th17 cells, is data showing that CD141^+^ cDC1s do not induce as many Th17 cells as CD1c^+^DCs.^6^ In addition, the cytokine IL-6, which we found to be involved in DC3 expansion and predominately produced by DC3s, is a dominant factor in the transcriptional program of Th17 cells and is imperative for the DC-mediated generation of pathogenic Th17 cells.^55^^,^^56^ The importance of these findings is underlined by studies reporting that Th1 and Th17 CD4 T cell subsets are crucial players in RA, are increased in SF and tissue, and are correlated with disease activity.^19^^,^^29^ PsA has been long considered a Th1 driven disease, however, subsequent research has highlighted the importance of Th17 cells in this disease. Both T cell subsets exert their pathological effects by secreting IFNγ and IL-17, thereby amplifying and sustaining chronic inflammation.^31^^,^^36^^,^^57^^,^^58^^,^^59^ Together, these studies and our assessment of the immune-stimulatory abilities of SF DCs suggest a role for DC3s in IA pathophysiology through priming Th17 and Th1 responses.

The SF-induced transformation of CD14^−^DC2s into CD14^+^CD163^+^ DC3s shows that DC3-like cells can arise from DC2s, acquiring the complete DC3 phenotype (CD1c^+^CD14^+^CD163^+^) in a stepwise manner. We observed that CD14 was the first to be upregulated by CD1c^+^ DCs, followed by CD163. All CD14^−^DC2s donors tested could acquire a DC3-like phenotype when stimulated with SF from various patients *in vitro*. In contrast, monocytes failed to sufficiently upregulate CD1c and did not acquire a DC3-like phenotype in the same setting. The monocytic lineage has long been considered a precursor to inflammatory DCs given their ability to develop into monocyte-derived dendritic cells (Mo-DCs) *in vitro*. However, the data presented here together with the pseudo-time analysis of CD5^+^ cDC2s to inflammatory DC3s from Dutertre et al.,^19^ as well as findings on DC3 development from Cytlak et al.*,*^21^ Bourdely et al.^20^ and Becker et al.*,*^18^ and the well-established distinction between Mo-DCs and DCs,^60^ confirms that inflammatory DCs are related to cDC2s rather than to monocytes. However, our findings differ from the findings of Cytlak et al.^21^ and Bourdely et al.,^20^ who report that DC3s and DC2s mostly develop from distinct progenitors. Bourdely et al.^20^ shows that DC3s can develop independently of common dendritic cell progenitor (CDPs), whereas DC2s develop mostly from CDPs.^20^ Importantly, in addition to an exclusive DC3 progenitor, they observed that most individual progenitors giving rise to DC3s had multi-lineage potential, of which a large fraction included DC2 potential.^20^ Cytlak et al.^21^ also show different developmental trajectories of DC2s and DC3s. DC2s developed from LMPPs (lymphoid-primed multipotent progenitor) along an IRF8^high^ pathway. DC3s developed predominantly, yet not exclusively, via CD33^+^GMP (granulocyte-macrophage progenitor) along an iRF8^low^ pathway.^21^ Our findings add a shared developmental trajectory between DC3s and DC2s to this, which potentially overlaps with the GMDP-containing fraction that Bourdely et al.^20^ reported. Of note, Bourdely et al.^20^ explored DC3 induction from DC2s using GM-CSF, which failed to induce CD14^+^ DCs. This is in line with our previous findings reported by Becker et al.^18^ and does not exclude DC2s as possible progenitors, as it is a matter of the DC3-promoting factors (IL-6, M-CSF, or PGE2) not being present.^18^ It is possible that under homeostatic conditions, there is a continuous influx of DC3s from the bone-marrow progenitors that increases under inflamed conditions, including an increased amount of DC2s developing into DC3s driven by cytokines. In summary, DC2s seem to contribute to the CD14^+^CD163^+^ DC3s, which are not derived from monocytes.

In our efforts to dissect which soluble mediators in SF induce DC3s, we observed that IL-6 inhibition reduced the frequencies of SF-induced DC3s. In agreement with our results, an abundance of IL-6 in SF is frequently reported for patients with inflammatory arthritis, with concentrations around 2–20 ng/mL for RA and slightly lower concentrations for psoriatic arthritis (1–12 ng/mL).^61^^,^^62^ The importance of IL-6 for disease pathology is stressed by the findings that the highest IL-6 levels occur during disease onset,^63^ and overall IL-6 levels correlate with disease activity and joint destruction.^62^^,^^64^^,^^65^^,^^66^ Herewith, we add the expansion of DC3s to the plethora of IL-6 mediated effects important for IA. The administration of tocilizumab, an IL-6R blocking antibody, could prevent this *in vitro*. Tocilizumab treatment *in vitro*, as expected, effectively prevented DC3 formation induced by SF from patients with high IL-6 levels, bringing DC3 frequencies close to background levels. It would be optimal for a future study to take blood or SF samples before and after tocilizumab treatment. In a small pilot of patients with (*n* = 5) or without tocilizumab (*n* = 5) treatment, we could not observe a difference in DC3 frequency in the blood (data not shown). The importance of investigating the interactions between tocilizumab and DCs expands beyond IA, as IL-6 is an important cytokine in, for instance, COVID-19 and several auto-immune diseases as well, diseases which are associated with an increased frequency of DC3s.^19^^,^^34^^,^^67^^,^^68^ Altogether, we show a dominant role for IL-6 in expanding DC3s and thereby expose a mechanism of how tocilizumab might affect the immune system of patients with IA.

Interestingly, we observed that recombinant IL-1β and TNFα prevent IL-6 dependent CD14 upregulation. IL-1β and TNF α are two pro-inflammatory cytokines known to induce the activation of DCs. In a previous study into DC3 in cancer, we showed that cDC2s from HDs lose their phenotypic plasticity to convert to CD14^+^DC3s after maturation is induced by activation stimuli.^18^ These findings most likely explain why IL-1β and TNF α also prevent IL-6 dependent CD14 upregulation in this study. Other contributing explanations can be sought in the findings that IL-1β and TNFα have both synergistic and antagonistic interactions with IL-6.^69^ For instance, in macrophages, TNF signaling can induce the expression of suppressor of cytokine signaling (SOCS3). SOCS3 is an inhibitor of the JAK/STAT3 signaling pathway and works by docking onto gp100 thereby inhibiting IL-6 signaling.^70^ IL-1β, on the other hand, can activate p38, which also inhibits the JAK signaling pathway^69^ This may represent a mechanism by which TNF-α and IL-1β counteract the effects of IL-6.

Finally, we directed our efforts to determine which pathway downstream of the IL-6R receptor is responsible for the conversion to DC3s induced by IL-6. Using small-molecule inhibitors of JAK/STAT3, we demonstrate an important role for JAK/STAT3 signaling in SF-induced DC3 development. JAK/STAT3 is activated by numerous cytokines, with IL-6 being the most prominent one. Upon binding of IL-6 to the IL-6R, STAT proteins are recruited to the complex, which are subsequently phosphorylated, leading to STAT3 dimers that can bind to STAT3-response DNA motifs.^50^ It is therefore not surprising that both pan JAK inhibitors and a STAT3 inhibitor had a notable effect on DC3 conversion induced by IL-6. The involvement of JAK/STAT3 signaling in arthritis is further supported by a study from Isomäki et al., showing upregulated STAT3 mRNA levels in blood and SF monocytes and T cells from patients with RA, which correlated with IL-6 plasma levels.^71^ Another study showed IL-6/JAK/STAT3 signaling was the main pathway induced by SF in monocytes in *in vitro* experiments.^4^ Moreover, recent research showed that IL-6/JAK/STAT3 signaling in immune cells correlates with disease progression in RA.^72^ Collectively, these studies suggest roles for JAK/STAT3 signaling in immune cells in IA. Herewith, we add DCs to this collection by showing a role for JAK inhibitors in the development of DC3, with potential implications for therapy in patients with IA.

In conclusion, our data support a dominant role for IL-6 and JAK/STAT3 signaling in the DC2 to DC3 development under inflammatory conditions. The performed enumeration, functional and phenotypic assessment of inflammatory DC3s, which suggests preferential Th1 and Th17 activation for SF DC3s, indicate an important role for DC3s in the disease activity and development of IA.

### Limitations of the study

In this study, we demonstrate that CD14^−^DC2s can convert to CD1c^+^CD14^+^CD163^+^ DCs driven by IL-6 in SF. Even though we observe similar effects of our *in vitro*-derived CD1c^+^CD14^+^CD163^+^ DC3s and *ex vivo* CD1c^+^CD14^+^CD163^+^ DC3s on T cells and a similar phenotype with CD14 and CD163 expression, we do not have transcriptional data to compare *in vitro-derived* DC3s with DC3s *in vivo*.

We investigated the frequency of CD1c^+^CD14^+^CD163^+^ DC3s in blood and SF of patients with IA. Since this study was started, additional markers such as CD88 and FCε RI have been discovered to better define DC3s and exclude monocytes. Our study did not use either of those markers. However, we performed additional stainings on PBMCs from HDs that showed no to minimal differences in the frequency of DC3s following our gating strategy with or without CD88 and FCε RI (not shown). We did similar measurements for CD56, as it has been shown that CD1c^+^DCs can express CD56.^73^ We observed that ∼3% of CD1c^+^DCs in PBMCs express CD56. Although the fraction of CD1c^+^DCs that are potentially excluded from our analysis because our lineage contained CD56 is minimal, we do recommend excluding CD56 and to include CD88 and FC ε RI in future DC subset studies.

Lastly, in recent years, the differences in the female and male immune systems have become more apparent. Therefore, it becomes more important to stratify patients based on their sex to enable investigating these differences. Unfortunately, due to the limited number of patient samples and ethical constraints, stratification by sex was not possible. Future studies into DC3s, as well as other immune cell subsets, will be required to understand sex-specific differences.

## Resource availability

### Lead contact

Further information and requests for resources and reagents should be directed and will be fulfilled by the lead contact, I. Jolanda de Vries (jolanda.devries@radboudumc.nl).

### Materials availability

This study did not generate new unique reagents**.**

### Data and code availability

• All raw data in this article will be shared by the lead contact upon request.
• This study does not report original code.
• Any additional information required to reanalyze the data reported in this article is available from the lead contact upon request.

## Acknowledgments

We thank all the patients who donated blood and SF for their participation in this study. This research was supported by a Radboudumc PhD grant to A.M.D.B (Nijmegen, Netherlands) and I.J.M.d.V. received Health Holland grants DC4Balance (LSHM18056-SGF) and ImmuneHealth-Seed (LSHM22042-SGF).

## Author contributions

Conceptualization, A.H.D, A.M.D.B, R.T., M.V., G.B., and I.J.M.d.V.; investigation, A.H.D, A.M.D.B., L.R.S., and T.v.O.; data analysis, A.H.D, and A.M.D.B.; resources – patient samples, L.L.v.d.H, C.D.P, R.M.T, and R.T.; writing – original draft, A.H.D and A.M.D.B, visualization and writing – review and editing, A.H.D, A.M.D.B, I.J.M.d.V, M.V, L.L.v.d.H, M.A.B, C.D.P, R.M.T, and R.T.; supervision, A.M.D.B., M.V., G.B., M.A.B., R.T., and I.J.M.d.V.; funding acquisition, A.M.D.B, M.V., R.T., and I.J.M.d.V.

## Declaration of interests

The authors declare no competing interests.

## STAR★Methods

### Key resources table

**Table:** 

REAGENT or RESOURCE	SOURCE	IDENTIFIER
**Antibodies**		

BV785 anti-human CD45 antibody, clone HI30	Biolegend	BioLegend Cat# 304048, RRID:AB_2563129
APC anti-human CD14 antibody, clone M5E2	Biolegend	BioLegend Cat# 301808, RRID:AB_314190
APC-H7 anti-human CD14 antibody, clone MϕP9	BD Biosciences	BD Biosciences Cat# 560180, RRID:AB_1645464
PE anti-human CD163 antibody, clone GHI/61	BD Biosciences	BD Biosciences Cat# 556018, RRID:AB_396296
BV421 anti-human CD1c antibody, clone L161	Biolegend	BioLegend Cat# 331526, RRID:AB_10962909
FITC anti-human CD1c, clone L161	Biolegend	BioLegend Cat# 331518, RRID:AB_2073403
PE anti-human CD1c, clone AD5-8E7	Miltenyi	Miltenyi Biotec Cat# 130-113-302, RRID:AB_2726081
APC anti-human CD197 (CCR7) antibody, clone	Miltenyi	Miltenyi Biotec Cat# 130-120-466, RRID:AB_2784047
FITC anti-human CD20 antibody, clone L27	BD Biosciences	BD Biosciences Cat# 345792, RRID:AB_2868818
Pacific blue anti-human CD20 antibody, clone 2H7	BioLegend	BioLegend Cat# 302320, RRID:AB_493651
FITC anti-human CD5 antibody, clone L17f12	eBioscience	Thermo Fisher Scientific Cat# 11-0058-42, RRID:AB_1944383
PerCP anti-human HLA-DR antibody, clone L243	Biolegend	BioLegend Cat# 307628, RRID:AB_893566
PE-Cy7 anti-human HLA-DR antibody, clone L243	BioLegend	BioLegend Cat# 307616, RRID:AB_493588
APC anti-human CD86 antibody, clone FUN-1	BD Biosciences	BD Biosciences Cat# 555660, RRID:AB_398608
PE anti-human CD45RA antibody, clone HI100	BioLegend	(BioLegend Cat# 304108, RRID:AB_314412)
PE-Cy7 anti human CD86 antibody, clone FUN-1	BD Biosciences	BD Biosciences Cat# 561128, RRID:AB_10563077
PE-Cy7 anti-human PD-L1 antibody, clone MIH1	BD Biosciences	BD Biosciences Cat# 558017, RRID:AB_396986
APC anti-human CD19 antibody, clone REA675	Miltenyi Biotec	Miltenyi Biotec Cat# 130-113-642, RRID:AB_2726195
Pacific bluer anti-human CD19, antibody, clone HIB19	BioLegend	BioLegend Cat# 302232, RRID:AB_2073118
APC anti-human CD3 antibody, clone UCHT1	Thermo Fisher Scientific	Thermo Fisher Scientific Cat# 17-0038-42, RRID:AB_10805861
BV510 anti-human CD3 antibody, clone SK7	BioLegend	BioLegend Cat# 344828, RRID:AB_2563704
Pacific blue anti-human CD3, clone UCHT1	BioLegend	BioLegend Cat# 300431, RRID:AB_1595437
APC anti-human CD56 antibody, clone NCAM16.2	BD Biosciences	BD Biosciences Cat# 341027, RRID:AB_2868759
Pacific blue anti-human CD56, clone MEM-188	BioLegend	BioLegend Cat# 304629, RRID:AB_2282499
FITC anti-human CD56, NCAM16.2	BD Biosciences	BD Biosciences Cat# 345811, RRID:2868832
AF647 anti-human IL-17 antibody, clone BL168	Biolegend	BioLegend Cat# 512310, RRID:AB_961388
Pacific blue anti-human CD66b, clone G10F5	BioLegend	BioLegend Cat# 305111, RRID:AB_2563293
PE anti-human IL-4 antibody, clone 7A3-3	Miltenyi Biotec	Miltenyi Biotec Cat# 130-091-647, RRID:AB_615125
FITC anti human IFNy antibody antibody, clone 4515	Miltenyi Biotec	Miltenyi Biotec Cat# 130-113-492, RRID:AB_244194
BV421 anti human IFNy antibody, clone B27	BD Biosciences	BD Biosciences Cat# 562988, RRID:AB_2737934
FITC anti-human antibody, clone 4515	Miltenyi Biotec	Miltenyi Biotec Cat#130-113-492, RRID:AB_244194
PerCP anti-human CD4 antibody, clone RPA-T4	Biolegend	BioLegend Cat# 300528, RRID:AB_893321
BV421 anti-human CD4 antibody, clone RPA-T4	BD Biosciences	BD Biosciences Cat# 562424, RRID:AB_11154417
PE anti human CD3 antibody, clone HIT3a	BD Biosciences	BD Biosciences Cat# 555340, RRID:AB_395746
BV421 anti-human CD3 antibody, clone SK7	BioLegend	BioLegend Cat# 344834, RRID:AB_2565675
FITC anti-human CD3 antibody, clone HIT3a	BD Biosciences	BD Biosciences Cat# 555339, RRID:AB_395745
FITC anti-human CD8, clone RPA-T8	BD BioSciences	BD Biosciences Cat# 555366, RRID:AB_395769
APC anti-human CD8, clone RPA-T8	BD BioSciences	BD Biosciences Cat# 555369, RRID:AB_398595
FITC anti human CD20 antibody, clone L27	BD Biosciences	BD Biosciences Cat# 345792, RRID:AB_2868818

**Biological samples**		

PBMCs from healthy donors (Buffycoat)	Sanquin, Nijmegen, The Netherlands	N/A
PBMCs from arthritis patients	Department of Tumor Immunology, Radboudumc, Nijmegen, The Netherlands	N/A
SFMC from arthritis patients	Department of Tumor Immunology, Radboudumc, Nijmegen, The Netherlands	N/A
Synovial fluid from arthritis patients	Department of Tumor Immunology, Radboudumc, Nijmegen, The Netherlands	N/A

**Chemicals, peptides, and recombinant proteins**		

E780 Fixable viability dye (1 in 1000)	ThermoFisher Scientific	Cat#65-0865-14
E506 Fixable viability dye (1 in 1000)	ThermoFisher Scientific	Cat#65-0866-14
CellTrace™ Violet (final conc 2.5μM)	ThermoFisher Scientific	Cat#C34557
CellTrace™CSFE (final conc 2.5μM)		
Tocilizumab (RoActemra)	Roche	EU/1/08/492
Sunitinib (Sutent)	Pfizer	EU/1/06/347/006
aEP2	Cayman Chemical, USA	Cat#AH6809
aEP4	Cayman Chemical, USA)	Cat#L-161982
Ultra-LEAF Purified Human IgG1 Isotype control (Clone QA16A12)	Biolegend	Cat#403502
Stattic	BioTechne	Cat#2789
Tofacitinib (CP-690550)	Selleckchem	Cat#S2789
Ruxolitinib (INCB018424)	Selleckchem	Cat#941678-49-5
IL-6	Miltenyi Biotec	Cat#130-093-933
TNFα	Miltenyi Biotec	Cat#130-094-014
IL-1β	Miltenyi Biotec	Cat#130-093-898

**Critical commercial assays**		

LEGENDplex™ Human Inflammation Panel 1 (13-plex)	Biolegend	Cat#740809
CD1c (BDCA-1)+Dendritic cell isolation kit, human	Miltneyi	Cat#130-119-475
Naive Pan T cell isolation kit, human	Miltenyi	Cat#130-097-095
Human IL-6 ELISA kit	ThermoFisher Scientific	Cat#KHC0061

**Software and algorithms**		

FlowJo V10	BD	https://www.flowjo.com
GraphPad Prism software (V8)	GraphPad Software	https://www.graphpad.com

### Experimental model and study participants details

Human peripheral blood mononuclear cells (PBMCs) were obtained from buffy coats from healthy donors that provided informed consent (Sanquin, Nijmegen). PBMCs from ERA, RA, HD-CI, JC cohorts were isolated from blood (TRI Brisbane). PBMCs from children with JIA were obtained from the Sint Maartenskliniek (Nijmegen, Netherlands) and isolated from blood, while PBMCs from healthy juveniles were handled in the Translational Research Institute in Brisbane (Australia). To confirm comparable procedures, independent examinations of PBMC samples from healthy adults were performed at both research institutes. For all cohorts, RA, OA, PsA and JIA was defined based on clinical diagnosis by rheumatologists. Additionally, for patients in the ERA cohort, DAS4-CRP formed by C-reactive protein levels, number of painful and swollen joint and patient’s general health measured on a visual analogue scale,^35^ is reported (Table S1). Patients in the ERA cohort were either untreated or had recently started treatment, all long-standing RA patients were treated for their RA. The use of human samples was approved by the Metro South HREC (94/QPAH 6) and the UQ research ethics committee (2012000275).

Arthritis patients for serum and blood collection were part of the “EXTRA study” and consisted of patients with active rheumatoid arthritis, psoriatic arthritis and peripheral spondyloarthritis (perSpA) all defined based on the clinical diagnosis by a rheumatologist and with all types of medication allowed (Table S2). Oral informed consent was obtained after a verbal explanation of the nature and scope of the research project, according to the protocol and applicable Dutch laws. Documentation of consent in the electronic patient records to the participation in the specific protocol was considered sufficient and approved by the Institutional Review Board (IRB) of the Radboudumc (CMO region Arnhem-Nijmegen; dossier 2015–1847) and conducted according to the Declaration of Helsinki. After informed consent, an extra blood sample was taken from these patients undergoing vena puncture for clinical diagnosis/monitoring.

All studies from which human material is included are conducted according to the principles of the Declaration of Helsinki and adhere to the Dutch Medical Research Involving Human Subjects Act (WMO) and Good Clinical Practice guidelines. Approval was granted by the Medical Ethical Committee Arnhem-Nijmegen (CMO). All participants provided informed consent. Patient information regarding age, sex, and disease status is listed in Tables S1, S2 and S3.

Leftover SF was obtained by joint aspiration from knees of patients attending the Sint Maartenskliniek (Table S3) as part of standard clinical care. Patients were either diagnosed with a known inflammatory arthritis with active disease or had new onset arthritis. Patients with suspected septic arthritis were excluded. Samples were also part of the “EXTRA study” (CMO region Arnhem-Nijmegen; dossier 2015–1847)

### Method details

#### Isolation of human blood immune cells

PBMCs were isolated using Lymphoprep (Axi-Shield PC AS). For CD14^+^ monocyte isolation, a fraction of PBMCs was incubated with CD14 MACS microbeads and Fc receptor (FcR) blocking reagent (Miltenyi), followed by isolation using LS columns (Miltenyi) according to the manufacturer’s protocol.

For isolation of CD14^-^DC2s, PBMCs were depleted for CD19 and CD14 using MACS microbeads and LD columns, followed by positive selection using the CD1c (BDCA1) DC isolation kit (Miltenyi) following the manufacturer’s protocol. Purity was assessed by flow cytometry and >95% for all samples (Figure S5B). To obtain the different DC2 subsets (CD1c^+^CD14^−^CD163^−^, CD1c^+^CD14^-^CD163^+^, CD1c^+^CD14^+^CD163^-^, CD1c^+^CD14^+^CD163^+^), PBMCs or SF cells were treated with the FCR blocking reagent and depleted for CD19, CD3 and CD56 sing MACS microbeads. Consequently, positive selection of CD1c was performed prior to staining with an appropriate sorting panel for 30 min at 4°C in sterile polypropylene round-bottom tubes (Falcon) (Table S5). Sorting was performed on a BD FACSMelody sorter (Gating strategy in (Figure S1B, Figure S2B). Allogeneic pan T cells and naïve T cells for T cell activation assays were isolated using a Pan T cells Isolation Kit (Miltenyi) or Naïve Pan T cell Isolation Kit (Miltenyi) (Figure S5A), respectively, according to the manufacturer’s protocol. Cultures with primary DC2s, monocytes and T cells were performed in X-VIVO™15 (Lonza) supplemented with 2% human serum (HS) (Sigma-Aldrich).

#### Processing of SF

Prior to any procedures with SF, SF samples were treated with 10 IU/ml hyaluronidase (Sigma H3506)^74^ for 30–40 min at 37°C followed by filtration over a 100 μM cell strainer (Corning). Cells were pelleted by centrifugation (10 min, 931 × g), and cleared SF was collected, aliquoted, and stored at -80°C until further use. Flow cytometry assessment was performed on freshly isolated SF cells. When the cell numbers were sufficient, DC2s were isolated from the remaining fraction of SF cells using MACS (described above), cryopreserved, or used immediately in allogeneic T cell assays. DC2s from SF were cultured in X-VIVO™15 (Lonza, Basel, Switzerland) supplemented with 2% HS.

#### Monocyte and DC2 conversion experiment

For all cell culture conversion experiments, 50.000–100.000 DC2s or monocytes were cultured in a total of 200 μL X-VIVO 2% HS, with or without 50% SF, in round-bottom 96-well plates for the indicated time points. To assess the effect of human recombinant cytokines, CD14^-^ DC2s were incubated with for 48h with cytokines (carrier free) in the following concentrations: 20ng/ml TNFα, 20ng/ml IL-1β and 1ng/ml IL-6. To inhibit the IL-6R, cells were pre-incubated with 10 μg/mL tocilizumab (RoActemra, Roche) for 30 min at 37°C and 5% CO_2_. To inhibit M-CSF and PGE2 signalling through EP2/EP4, cells were pre-incubated with 100nM sunitinib or soluble aEP2/4 (30μM of aEP2 and 5μM of aEP4) respectively. To inhibit JAK/STAT signalling, cells were pre-incubated with 0.2, 0.5, or 1 μM Ruxolitinib (Selleckchem S1378), 1 μM or 2 μM Tofacitinib (Selleckchem S2789) or 1 μM of Stattic (BioTechne 2789) for 1 h at 37°C and 5% CO_2_. Control samples were incubated with 10 μg/mL Ultra-LEAF purified human IgG1 isotype control (clone QA16A2, BioLegend) and DMSO as vehicle control. After pre-incubation, 100 μL SF (50%) was added to the cells. After a culture period of 48 h, cells were harvested and analysed by flow cytometry (described below). When sorting was required for functional assays after the conversion experiment, CD14^−^ cD2s were cultured at a concentration of 0.75·10^6^ cells/mL in round-bottom polypropylene tubes (cat: 352059) containing 60% X-VIVO 2% HS supplemented with 40% SF. After 36 h, the cells were harvested and prepared for sorting (Tables S4 and S5). Sorting was performed on a BD FACSMelody sorter.

#### Human cell flow cytometry

Antibodies used for flow cytometry with corresponding dilutions are listed in Table S4 and panels used in Table S5. Gating strategies are shown in Figure S1A, S1B, S1C, S2A, S2B, S2E, S3A. Briefly, flow cytometry stainings were performed in V-bottom 96 well plates (Greiner Bio-one) at 4°C protected from light. Cells were washed and incubated with the Fixable Aqua dead cell stain kit (Invitrogen) or eBioscience Fixable Viability Dye e506 or e780 (Thermo) for 25 min in PBS, washed, and incubated for 15 min with FcR blocking reagent (Miltenyi). For all intracellular staining, cells were fixed and permeabilized with BD Cytofix/Cytoperm (BD BioSciences) according to the manufacturer’s instructions. All stainings were performed with directly labelled primary antibodies for 30 min at 4°C protected from light, after which cells were washed twice more prior to acquisition. Anti-mouse Ig, κ/Negative Control Compensation Particles Set (BD), and AbC™ Total Antibody compensation kit (Invitrogen) were used as single stain controls. Flow Cytometry acquisition was performed on a LSR Fortessa, BD FACSverse or BD FACSLyric and Flowjo v.10 (Tree Star) was used to analyse data.

#### ELISA

SF samples were analysed with the IL-6 ELISA kit from Invitrogen, following the manufacturer’s instructions. Samples were diluted 1:10, 1:100, 1:500, 1:1000, 1:2000, 1:4000, 1:8000. Sample acquisition was performed on the iMark™ Microplate reader (BioRad), and cytokine concentrations were calculated relative to the standard curve in Excel software. For analysis appropriate dilution per donor were utilised. The mean value was calculated from the measurements of the dilutions that fell within the detectable range.

#### Cytokine quantification

Sorted DCs, as described previously, were cultured in 100 μL medium for 21 h. Cultured supernatants were collected and stored at -20°C until analysis by the LEGENDplex ™ Human inflammation panel 1 (BioLegend). LEGENDplex™ was performed according to the manufacturer’s instructions, acquired on the MACSQuant Analyzer, and data were analysed using the LEGENDplex™ Data analysis Software (Table S6).

#### Allogenic T cell assays

DC2s were isolated as described above and cultured overnight in the absence or presence of 20 μg/mL poly I:C (Invivogen) and 4 μg/mL R848 (Invivogen). To assess proliferation and polarization, allogenic naïve pan T cells were labelled with 2.5 μM CellTrace CFSE or Violet (CTV) dye (Thermo Fisher) for 10 min at 37°C. For proliferation without polarization analysis, a total of 6 000 cells from sorted DC subsets were co-cultured with 30 000 CFSE-labelled allogeneic Pan T cells for five days. Cells were harvested and stained for membrane markers (described above) and acquired on a flow cytometer followed by analysis in FlowJo to determine the frequency of proliferating CD4^+^ and CD8^+^ T cells. For polarization, 6 000 DCs were co-cultured with 60 000 CTV-labelled allogenic naïve Pan T cells in a round-bottom plate in X-VIVO™15 (Lonza) supplemented with 2% HS for 6 days. On day 6, cells were stimulated with 25 ng/mL phorbol myristate and 0.5 μg/mL ionomycin (Merck) for 1 h at 37°C followed by 10 μg/mL Brefeldin A (Cayman Chemical) for an additional3 h. After incubation, cells were harvested and stained for membrane markers and intracellular cytokines (as described above).

### Quantification and statistical analysis

Statistical analysis was performed using GraphPad Prism software (V8), unless otherwise indicated. Results are presented as mean±SD, unless otherwise indicated in the figure legends. For multiple group comparisons, one-way or two-way analysis of variance (ANOVA) or mixed-effects analysis was performed, followed by correction for multiple testing as indicated in each figure legend. Significance between two groups was tested using the paired or unpaired Student’s t-test. Statistical significance was annotated as ∗*p* < 0.05, ∗∗*p* < 0.01, ∗∗∗*p* < 0.001, ∗∗∗∗*p* < 0.0001.
