# Supplementary material for: Interleukin-6 in synovial fluid drives the conversion of DC2s to DC3s in inflammatory arthritis
Source: iScience. 2025 Jun 20;28(7):112957. doi: 10.1016/j.isci.2025.112957 (PMC12270937; doi:10.1016/j.isci.2025.112957)
Supplement: Document S1. Figure S1–S5 and Tables S1–S6 [file mmc1.pdf]

iScience, Volume *28*

Supplemental information

Interleukin-6 in synovial fluid drives the conversion of DC2s to DC3s in inflammatory arthritis

Annika H. Decker, Lucas L. van den Hoogen, Tom van Oorschot, Liliana Sanchez-Rocha, Martine A. Boks, Ghaith Bakdash, Ranjeny Thomas, Calin D. Popa, Martijn Verdoes, Rogier M. Thurlings, Anouk M.D. Becker, and I. Jolanda M. de Vries

Supplementary figures and tables

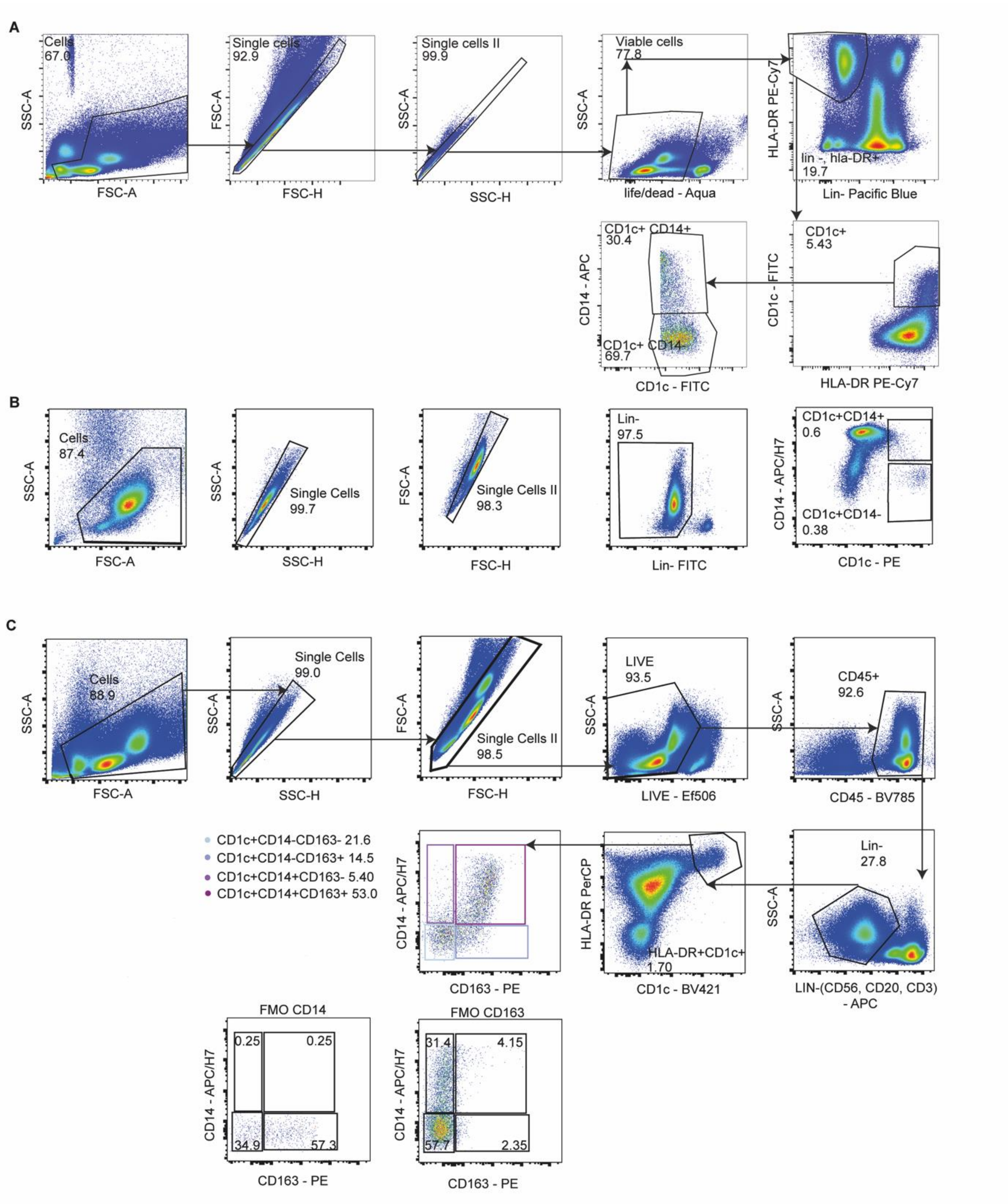

Figure S1. Gating strategy for enumeration and isolation of DC2s from peripheral blood

(A) Gating strategy for the enumeration of $CD14^+$ DC3s and $CD14^-$ DC2s in peripheral blood of arthritis patients and healthy donor cohorts (corresponds to Figure 1A-C). **(B)** Gating strategy to isolate $CD14^-$ DC2s and $CD14^+$ DC3s from peripheral blood of arthritis patients. After PBMCs were depleted for CD3, CD19, and CD56 lineage (LIN) positive cells using MACS microbeads, cells were stained with the appropriate sorting panel and sorted on a BD FACSMelody (corresponds to Figure 1 D-E). **(C)** Gating strategy for the enumeration of $CD1c^+CD14^+CD163^+$ DC3s in peripheral blood of arthritis patients (corresponds to Figure 1F).

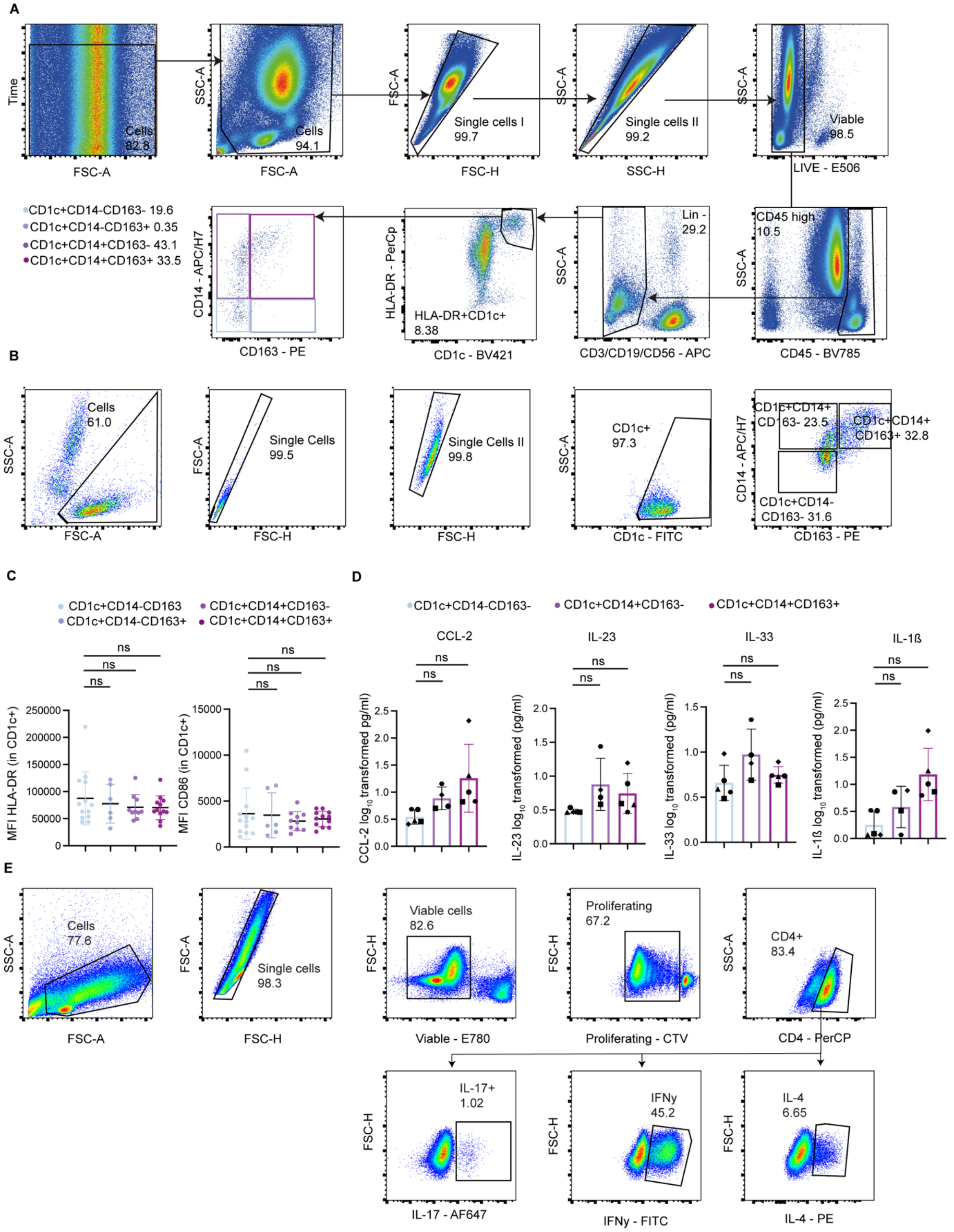

Figure S2. Gating strategy for enumeration and isolation of CD1c⁺ DCs from synovial fluid of arthritis patients for functional assays

(A) Gating strategy for synovial fluid samples performed on fresh material directly after hyaluronidase treatments (corresponds to Figure 2B, S2C). **(B)** Gating strategy to isolate three different CD1c⁺DC subsets from synovial fluid. After lineage negative depletion, CD1c positive cells were selected using

the CD1c (BDCA1) DC isolation kit. Cells were stained with the appropriate sorting panel and sorted on a BD FACSMelody (corresponds to Figure 2C-D). **(C)** Phenotype of CD1c$^+$ DC subsets in synovial fluid of patients with arthritis based on CD14 and CD163 expression. gFMI; median fluorescence intensity. Each dot represents one synovial fluid donor (mean ± SD, n=13, one-way-ANOVA followed with Tukey's test). **(D)** CD1c$^+$ DCs were FACS sorted into three subsets based on CD163 and CD14 expression after CD1c$^+$ isolation from synovial fluid. Sorted DCs were cultured for 21h and cytokine secretion measured using the LEGENDplex Human inflammation panel (mean ± SD, n = 5, each the mean of technical replicates). Significance was tested with a one-way ANOVA, Turkey's multiple comparison test. *p<0.05, **p<0.01, ***p<0.001, ****p<0.0001. **(E)** Gating strategy for CTV labelled naïve T cells after co-culture of 6 days with sorted DC subsets (corresponds to Figure 2D).

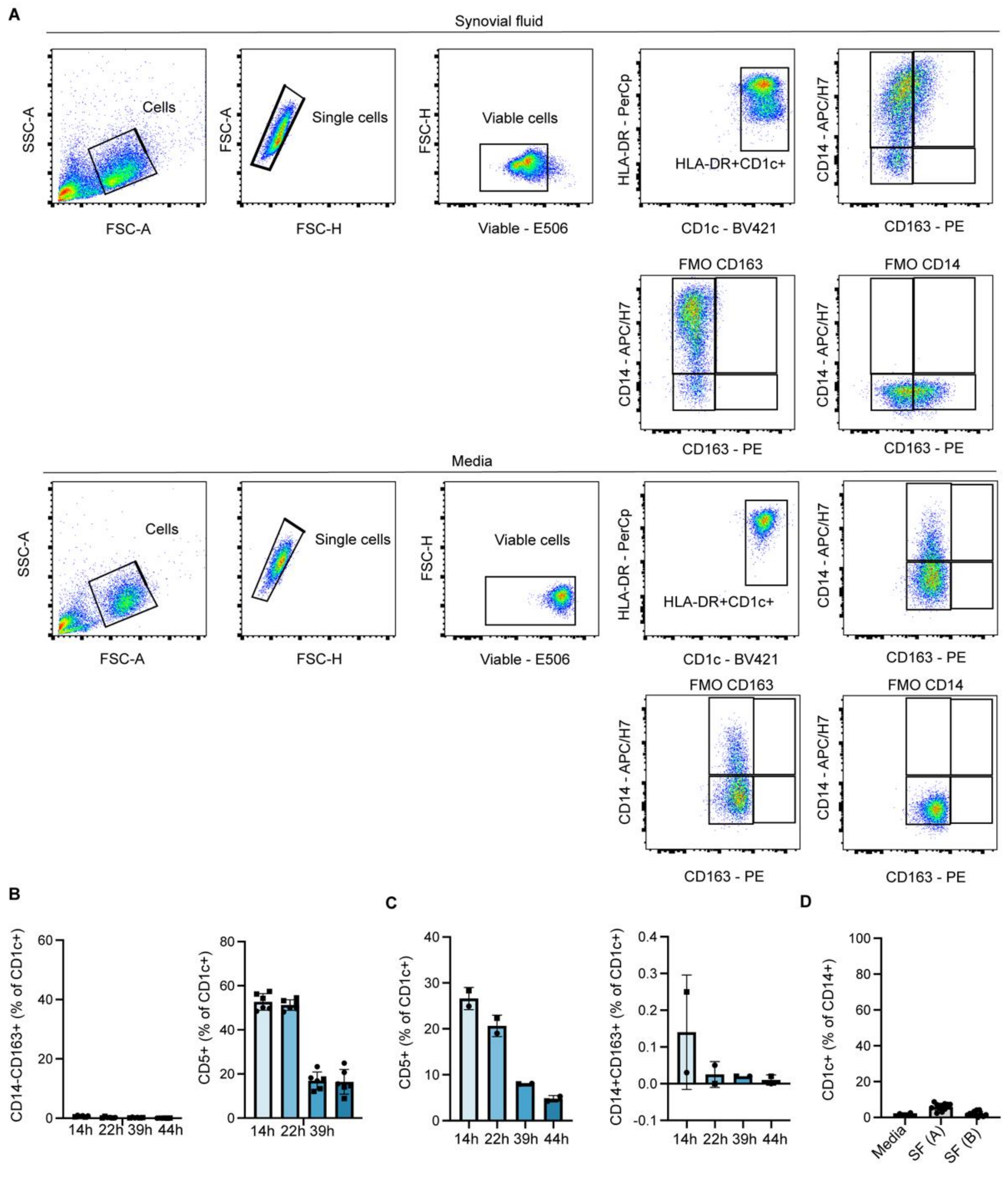

Figure S3. Gating strategy and phenotypical characterisation for DC3 induction experiment from CD14$^-$DC2s

(A) Gating strategy of CD14$^-$DC2s exposed to 50% synovial fluid (SF) or media for 44 h. After two days the cultured cells were analysed by flow cytometry for CD14 and CD163 expression with FMOs used for identifying gating boundaries (corresponds to Figure 3B-C). **(B)** Frequencies of CD14$^-$CD163$^+$ DCs

and CD5$^+$ DCs after isolation of CD14$^-$DC2s from blood of healthy donors (n=2) depicted with square and dot and cultured with 50% hyaluronidase-treated SF from arthritis patients (n=3) for 14h, 21h, 39h and 44h (mean ± SD). **(C)** Frequency of CD1c$^+$CD14$^+$C163$^+$ DC3s and CD5$^+$ DCs after isolation of CD14$^-$ DC2s from blood of healthy donors (n=2) depicted with squares and dots and cultured with medium for 14h, 21h, 29h and 44h (mean ± SD). **(D)** CD14$^+$ monocytes were isolated from blood of healthy donors (n=2) and cultured with hyaluronidase-treated synovial fluid from arthritis patients for two days (n=10)**.**

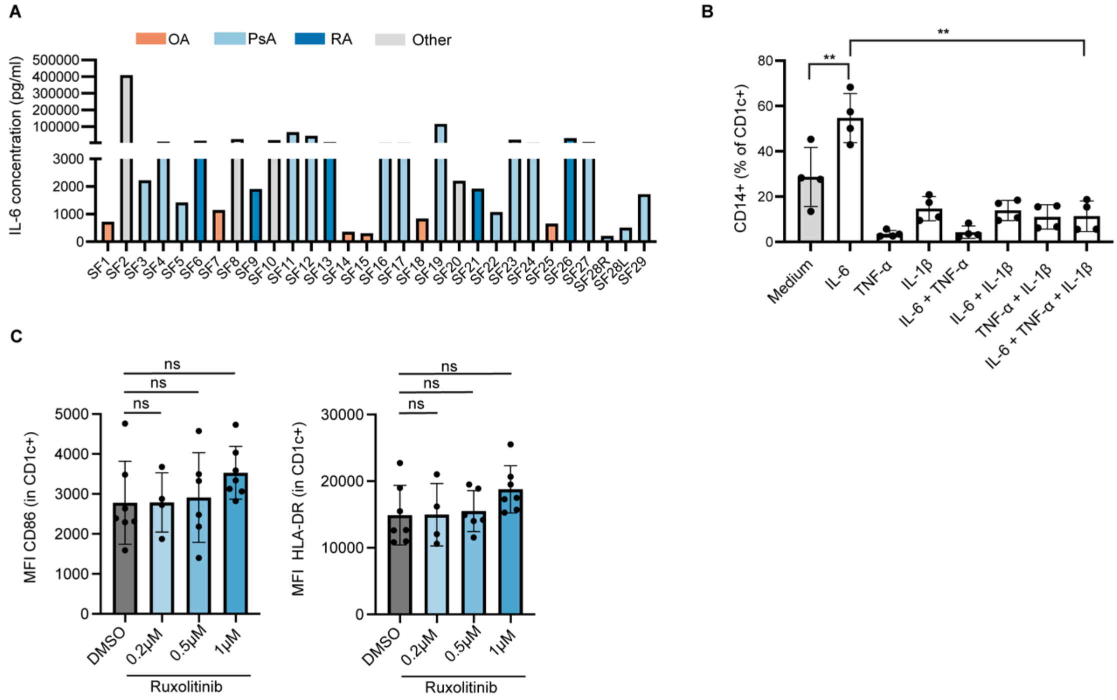

Figure S4. DC3 induction experiment from CD14$^-$DC2s

(A) Concentration of IL-6 in SF determined by ELISA (n=27 different patients, with SF of patient 28 right (R) and left (L) knee and SF16 and SF23 being the same patient) **(B)** Effect of two-day culture with recombinant human cytokines on CD14$^+$ DC2 induction compared to medium. Each symbol represents a biological replicate (n=4, mean ± SD), asterisks show significant results compared with medium. Significance was tested with a one-way ANOVA, Turkey's multiple comparison test. **(C)** CD14$^-$ DC2s were isolated from blood of healthy donors and cultured with 50% hyaluronidase-treated synovial fluid from arthritis patients and expression of HLA-DR and CD86 was measured in the presence or absence of varying concentrations of Ruxolitinib by flow cytometry. Each dot represents a biological replicate (mean ± SD, n =5). Significance was tested with a one-way ANOVA, followed by Dunnett's multiple comparison test versus DMSO control. *p<0.05, **p<0.01, ***p<0.001, ****p<0.0001. MFI; median fluorescence intensity.

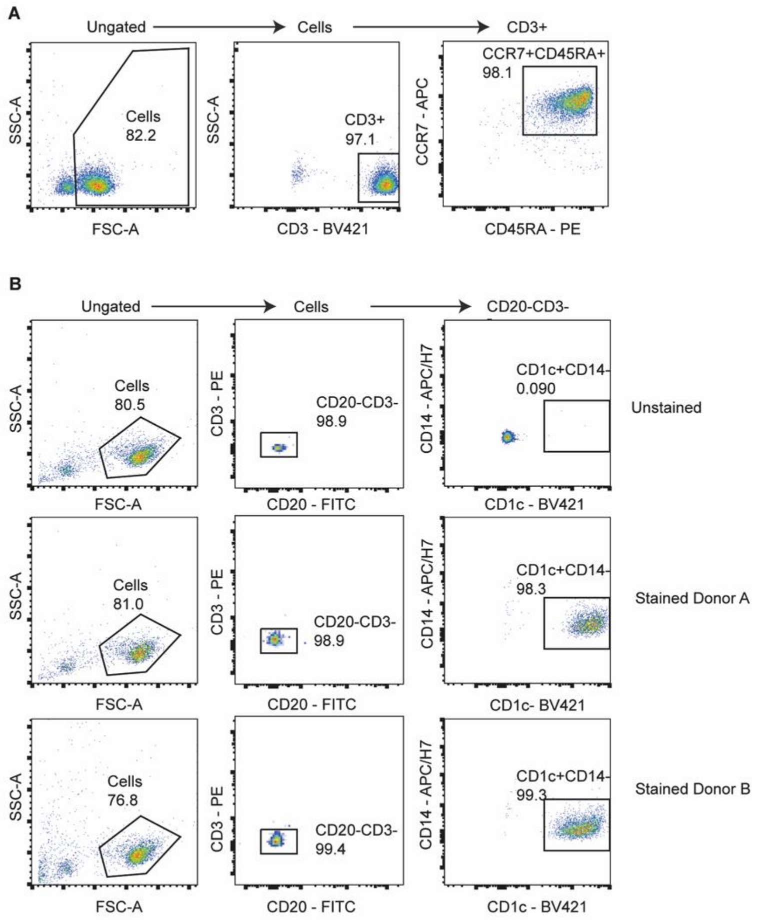

Figure S5. Purity analysis of DCs and naïve T cells
(A) Purity was analysed by flow cytometry after isolation of naive Pan T cells using MACS microbeads (corresponds to Figure 2D, 3E). See also table S4,S5. **(B)** Purity analysis by flow cytometry after isolation of $CD14^{-}CD1c^{+}$ DCs. After MACS depletion of $CD19^{+}$ and $CD14^{+}$ cells, $CD1c^{+}$ cells were isolated by positive selection using CD1c (BDCA1) DC isolation kit (Miltenyi). Representative dot plots of A) unstained sample) and B) two representative donors for $CD1c^{+}CD14^{-}$ cells, all stained with primary directly labelled antibodies: CD1c-BV421, CD20-FITC, CD3-PE ad CD14-APC/H7 and assessed by flow cytometry (corresponds to Figure 3-5). See also table S4, S5.

Table S1. Patient information related to Figure 1A-C, Figure S1AB

ID	disease	DAS4-CRP	sex	age
1279	Rheumatoid arthritis (early stage)	6,568288	M	60
1949	Rheumatoid arthritis (early stage)	2,441081	M	21
3169	Rheumatoid arthritis (early stage)	1,819955	F	49
3570	Rheumatoid arthritis (early stage)	1,693517	M	31
3992	Rheumatoid arthritis (early stage)	1,745033	F	51
4520	Rheumatoid arthritis (early stage)	2,861429	F	57
7189	Rheumatoid arthritis (early stage)	4,022095	F	68
8618	Rheumatoid arthritis (early stage)	2,540475	F	64
R1163	Rheumatoid arthritis (long established)	n/a	M	62
R1959	Rheumatoid arthritis (long established)	n/a	F	26
R2647	Rheumatoid arthritis (long established)	n/a	F	44
R2652	Rheumatoid arthritis (long established)	n/a	F	77
R3000	Rheumatoid arthritis (long established)	n/a	F	66
R3954	Rheumatoid arthritis (long established)	n/a	F	44
H1082	Healthy donor - cohort I (Brisbane)	-	F	33
H2048	Healthy donor - cohort I (Brisbane)	-	F	38
H2279	Healthy donor - cohort I (Brisbane)	-	M	69
H3634	Healthy donor - cohort I (Brisbane)	-	F	41
H4239	Healthy donor - cohort I (Brisbane)	-	F	47
H4421	Healthy donor - cohort I (Brisbane)	-	F	64
H4774	Healthy donor - cohort I (Brisbane)	-	F	29
pat 5	Juvenile idiopathic arthritis	n/a	F	13
pat 4	Juvenile idiopathic arthritis	n/a	F	6
pat 7	Juvenile idiopathic arthritis	n/a	F	15
pat 8	Juvenile idiopathic arthritis	n/a	F	17
pat 13	Juvenile idiopathic arthritis	n/a	M	12
pat 10	Juvenile idiopathic arthritis	n/a	F	7
pat 14	Juvenile idiopathic arthritis	n/a	M	10
pat 16	Juvenile idiopathic arthritis	n/a	M	16
	mean (sd)			10.75 (5.51)
	Healthy juveniles cohort n=3	-	n/a	4.1-0.4(mean-SD)

H5989	Healthy donor - juvenile			
H8981	Healthy donor - juvenile			
H5938	Healthy donor - juvenile			
	Healthy donor - cohort II (Nijmegen)			

Table S2. Patient information relaed to Figure 1D,F, Figure S1B,C

ID	disease	sex	age	clinical information	Treatment
**1**	Spondyloarthritis	F	21		infliximab
**2**	Spondyloarthritis	M	47		brodalumab, high dose prednison
**3**	Rheumatoid arthritis	M	52	erosive, rheumatoid factor and anti-CPP positive	methotrexaat, hydroxychloroquine
**4**	Rheumatoid arthritis	M	74		methotrexaat, sulfasalazine en hydroxychloroquine
**5**	Psoriatic arthritis	F	57		infliximab, prednisone
**6**	Rheumatoid arthritis	F	56	n/a	methotrexaat, adalimumab
**7**	Rheumatoid arthritis	F	61	n/a	none
**8**	Rheumatoid arthritis	M	59	n/a	methotrexaat, adalimumab

Table S3. Patient information related to Figure 2-5, Figure S2-4. Abbreviations: n: number; p50: median or percentile 50; p25-p75: range between percentiles 25 and 75; RA: Rheumatoid arthritis; OA: Osteoarthritis, PsA: Psoriatic arthritis, UA: Undiffeentiated arthritis; CRP: C-reactive protein

	**Disease**			
	**OA**	**PsA**	**RA/UA**	**Other arthritis***
**Disease, n**	6	12	4/1	4
**Female, n(%)**	3 (50%)	7 (58.33%)	4 (80%)	1 (25%)
**CRP**	1, [1-4.5]	5.5 [2.5-55]	15 [11-36,75]	105 [21-114]
**Age p50 [p25-p75)**	68 [56-72,5]	55,5 [42.25-66]	31 [23,5-64,5]	43,5 [29,25-59.25]
**Immunosuppressive treatment, n (%)**				
**Prednisone**		1 (8.33%)		2 (50%)
**Upadacitinib**		1 (8.33%)		
**Sulfasalazin**			1 (20%)	1 (25%)
**MTX/bariticinib**			1 (20%)	
**MTX**		3 (25%)	1 (20%)	
**Infliximab/prednisone**		1 (8.33%)		
**MTX/etanercept**			1 (20%)	
**MTX/rituximab**		1 (8.33%)		
**None**	6 (100%)	5 (41,67%)	1 (20%)	1 (25%)
*Reactive arthritis, Calcium Pyrophosphate Deposition (CPPD), Inflammatory bowel disease (IBD) associated arthritis, Peripheral SpA				

Table S4. Antibodies with corresponding dilutions used in this study. Related to Figures 1-5, Figures S1-5

Marker	Fluorochrome	Clone	Dilution	Source	RRID
anti-human CD14	APC	M5E2	1 in 40	Biolegend	(BioLegend Cat# 301808, RRID:AB_314190)
anti-human CD14	APC-H7	MφP9	1 in 30	BD Biosciences	(BD Biosciences Cat# 560180, RRID:AB_1645464)
anti-human CD163	PE	GHI/61	1 in 25	BD Biosciences	(BD Biosciences Cat# 556018, RRID:AB_396296)
anti-human CD19	Pacific Blue	HIB19	1 in 50	Biolegend	(BioLegend Cat# 302232, RRID:AB_2073118)
anti-human CD19	APC	REA675	1 in 50	Miltenyi	(Miltenyi Biotec Cat# 130-113-642, RRID:AB_2726195)
anti-human CD1c	FITC	L161	1 in 40	Biolegend	(BioLegend Cat# 331518, RRID:AB_2073403)
anti-human CD1c	BV421	L161	1 in 30	Biolegend	(BioLegend Cat# 331526, RRID:AB_10962909)
anti-human CD1c	PE	AD5-8E7	1 in 50	Miltenyi	(Miltenyi Biotec Cat# 130-113-302, RRID:AB_2726081)
anti-human CD20	Pacific Blue	2H7	1 in 50	Biolegend	(BioLegend Cat# 302320, RRID:AB_493651)
anti-human CD20	FITC	L27	1 in 10	BD Biosciences	(BD Biosciences Cat# 345792, RRID:AB_2868818)
anti-human CD3	Pacific Blue	UCHT1	1 in 250	Biolegend	(BioLegend Cat# 300431, RRID:AB_1595437)
anti-human CD3	BV510	SK7	1 in 50	Biolegend	(BioLegend Cat# 344828, RRID:AB_2563704)
anti-human CD3	APC	UCHT1	1 in 10	eBioscience	(Thermo Fisher Scientific Cat# 17-0038-42, RRID:AB_10805861)
anti-human CD3	PE	HIT3a	1 in 25	BD Biosciences	(BD Biosciences Cat# 555340, RRID:AB_395746)
anti-human CD3	BV421	SK7	1 in 20	BioLegend	(BioLegend Cat# 344834, RRID:AB_2565675)
anti-human CD3	FITC	HIT3a	1 in 20	BD Biosciences	(BD Biosciences Cat# 555339, RRID:AB_395745)
anti-human CD4	PerCP	RPA-T4	1 in 25	Biolegend	(BioLegend Cat# 300528, RRID:AB_893321)
anti-human CD4	BV421	RPA-T4	1 in 50	BD Biosciences	(BD Biosciences Cat# 562424, RRID:AB_11154417)
anti-human CD45	BV785	HI30	1 in 25	Biolegend	(BioLegend Cat# 304048, RRID:AB_2563129)
anti-human CD5	FITC	L17f12	1 in 100	eBioscience	(Thermo Fisher Scientific Cat# 11-0058-42, RRID:AB_1944383)
anti-human CD56	Pacific Blue	MEM-188	1 in 200	Biolegend	(BioLegend Cat# 304629, RRID:AB_2282499)

anti-human CD56	APC	NCAM 16.2	1 in 10	BD Biosciences	(BD Biosciences Cat# 341027, RRID:AB_2868759)
anti-human CD56	FITC	NCAM 16.2	1 in 10	BD Biosciences	(BD Biosciences Cat# 345811, RRID:2868832)
anti-human CD66b	Pacific Blue	G10F5	1 in 200	Biolegend	(BioLegend Cat# 305111, RRID:AB_2563293)
anti-human CD8	FITC	RPA-T8	1 in 33	BD Biosciences	(BD Biosciences Cat# 555366, RRID:AB_395769)
anti-human CD8	APC	RPA-T8	1 in 50	BD Biosciences	(BD Biosciences Cat# 555369, RRID:AB_398595)
anti-human CD86	PE-Cy7	FUN-1	1 in 20	BD Biosciences	(BD Biosciences Cat# 561128, RRID:AB_10563077)
anti-human CD86	APC	FUN-1	1 in 20	BD Biosciences	(BD Biosciences Cat# 555660, RRID:AB_398608)
anti-human HLA-DR	PE-Cy7	L243	1 in 100	Biolegend	(BioLegend Cat# 307616, RRID:AB_493588)
anti-human HLA-DR	PerCP	L243	1 in 30	Biolegend	(BioLegend Cat# 307628, RRID:AB_893566)
anti-human IFNγ	BV421	B27	1 in 50	BD Biosciences	(BD Biosciences Cat# 562988, RRID:AB_2737934)
anti-human IFNy	FITC	4515	1 in 50	Miltenyi	(Miltenyi Biotec Cat#130-113-492, RRID:AB_244194
anti-human IL-17	AF647	BL168	1 in 25	Biolegend	(BioLegend Cat# 512310, RRID:AB_961388)
anti-human IL-4	PE	7A3-3	1 in 25	Miltenyi	(Miltenyi Biotec Cat# 130-091-647, RRID:AB_615125)
anti-human PD-L1	PE-Cy7	MIH1	1 in 20	BD Biosciences	(BD Biosciences Cat# 558017, RRID:AB_396986)
anti-human CD45RA	PE	HI100	1 in 5	BioLegend	(BioLegend Cat# 304108, RRID:AB_314412)
anti-human CD197 (CCR7)	APC	REA546	1 in 50	Miltenyi	(Miltenyi Biotec Cat# 130-120-466, RRID:AB_2784047)

Table S5. Antibody panels used in this study. Related to Figures 1-5, Figure S1-5

		**Laser**	Violet (405nm)	Violet (405nm)	Violet (405nm)	Blue (488nm)	Blue (488nm)	Blue (488nm)	Blue (488nm)	Red (633nm)	Red (633nm)
		**Band pass filter#**	448/45 - 450/50	528/45 - 525/50	755 LP	527/32 - 530/30	586/42	700/54	783/56 - 780/60	660/10 - 670/14	783/56
	**Data in:**	**Cytometer**									
cDC2 frequency peripheral blood (Brisbane)	Figure 1A-C	LSR Fortessa	CD3/CD19/CD20/CD56/CD66b Pacific Blue	L/D Aqua		CD1c FITC			HLA-DR PE-Cy7	CD14 APC	
T cell proliferation panel	Figure 1D-E, Figure 2D	Lyric	CD4 BV421	CD3 BV510		CFSE				CD8 APC	L/D E780
DC sorting panel peripheral blood	Figure 1D-E, Figure S1B	Melody				CD3/CD20/CD56 FITC	CD1c PE				CD14 APC-H7
cDC2 frequency peripheral blood (Radboud)	Figure 1F, Figure S1C	Lyric	CD1c BV421	L/D E506	CD45 BV785	CD5 FITC	CD163 PE	HLA-DR PerCP	CD86 PE-Cy7	CD3/CD19/CD56 APC	CD14 APC-H7
cDC2 frequency synovial fluid	Figure 2B, Figure S2A Figure S2C	Lyric	CD1c BV421	L/D E506	CD45 BV785	CD5 FITC	CD163 PE	HLA-DR PerCP	CD86 PE-Cy7	CD3/CD19/CD56 APC	CD14 APC-H7
Sorting panel for DCs from SF	Figure 2C,D, Figure 3E, Figure S2B,D	Melody				CD1c FITC	CD163 PE				CD14 APC-H7
CD4 polarization (1 donor)	Figure 2D	Lyric	IFNγ BV421				IL-4 PE	CD4 PerCP		IL-17 AF647	L/D E780
CD4 polarization (4 donors)	Figure 2D, Figure 3E, Figure S2E	Lyric	Proliferation/Cell trace violet			IFNy FITC	IL-4 PE	CD4 PerCP		IL-17 AF647	L/D E780
Monocyte cultured with SF	Figure S3D	Lyric	CD1c BV421	L/D E506		CD20 FITC	CD163 PE	HLA-DR PerCP		CD86 APC	CD14 APC-H7

cDC2 cultured with SF	Figure 3B,C,D Figure S3A-C	Lyric	CD1c BV421	L/D E506	CD5 FITC	CD163 PE	HLA-DR PerCP		CD86 APC	CD14 APC-H7
cDC2 modulation	Figure 4C-G Figure 5 A-E	Lyric	CD1c BV421	L/D E506	CD5 FITC	CD163 PE	HLA-DR PerCP	(PD-L1 PE-Cy7)	CD86 APC	CD14 APC-H7
DC purity after isolation	Figure S5A	Lyric	CD1c BV421		CD20 FITC	CD3 PE				CD14 APC-H7
Naïve T cell purity after isolation	Figure S5B	Lyric	CD3 BV421			CD45RA PE			CCR7 APC	

LSR Fortessa filters are second filter in each cell
*Intracellular staining are italized and in green
L/D: life dead

Table S6. Cytokine secretion overall and per donor raw values, related to Figure 2C, Figure S2D

Average all donors													
	**IL-1β**	**IFNγ**	**IFN-α2**	**TNFα**	**MCP-1**	**IL-6**	**IL-8**	**IL-10**	**IL-12p70**	**IL-17**	**IL-18**	**IL-23**	**IL-33**
**Lower range**	<1.17	<0.40	<0.724	<2.96	<2.57	<0.41	<0.70	<0,65	<0.43	<0.101	<2.29	<2.93	<2.8
**CD14-CD163-DCs**	2,64	0,49	1,2	4,06	3,92	0,69	2134,25	1,39	1,03	0,12	12,86	3,48	5,12
**CD14+CD163-DCs**	6,11	0,56	2,4	29,78	8,52	22,33	9615	17,49	722,22	0,15	28,83	11,62	11,25
**CD14+CD163+DCs**	27,96	0,6	2,03	41,7	50,69	204,06	8836,8	60,23	1,85	0,17	22,22	6,26	5,77
**SF1**													
	**IL-1β**	**IFNγ**	**IFN-α2**	**TNFα**	**MCP-1**	**IL-6**	**IL-8**	**IL-10**	**IL-12p70**	**IL-17**	**IL-18**	**IL-23**	**IL-33**
**Lower range**	<1.17	<0.40	<0.724	<2.96	<2.57	<0.41	<0.70	<0,65	<0.43	<0.101	<2.29	<2.93	<2.8
**CD14-CD163-DCs**	3,26	0,49	1,295	4,06	4,81	0,71	6952,2	1,53	1,12	0.14	22,41	4,03	4,85
**CD14+CD163-DCs**	7,27	0,54	4	44,63	5,51	27,4	12000	29,8	1,63	0,2	28,18	8.12	9.35
**CD14+CD163+DCs**	10,35	0,59	2,36	75,83	10,38	98.44	12000	88,21	1,6	0,2	32.53	7.82	5.75
**SF2**													
	**IL-1β**	**IFNγ**	**IFN-α2**	**TNFα**	**MCP-1**	**IL-6**	**IL-8**	**IL-10**	**IL-12p70**	**IL-17**	**IL-18**	**IL-23**	**IL-33**
**Lower range**	<1.17	<0.40	<0.724	<2.96	<2.57	<0.41	<0.70	<0,65	<0.43	<0.101	<2.29	<2.93	<2.8
**CD14-CD163-DCs**	1,4	0,45	0,78	<2.96	3,04	0,67	875,12	1,01	0,6	0,12	7,59	2,93	3,65
**CD14+CD163-DCs**	3,38	0,49	1	38,01	5,32	13,48	4845,13	3,9	1,34	0,14	17,59	4,56	4,95
**CD14+CD163+DCs**	7,37	0,49	1,31	74,8	6.67	55,31	6597,75	13,08	2.50	0,15	17,12	4,68	4,4
**SF3**													
	**IL-1β**	**IFNγ**	**IFN-α2**	**TNFα**	**MCP-1**	**IL-6**	**IL-8**	**IL-10**	**IL-12p70**	**IL-17**	**IL-18**	**IL-23**	**IL-33**
**Lower range**	<1.17		<0.724	<2.96	<2.57	<0.41	<0.70	<0,65	<0.43	<0.101	<2.29	<2.93	<2.8

**CD14-CD163-DCs**	1,17	0,5	1,52	<2.96	2,99	<0.41	284,72	2,31	1,85	0,14	19,27	<2,93	3,9
**CD14+CD163+DCs**	17.91	0,55	1,76	12,02	20,46	41,53	7069,1	4,62	1,09	0,11	10,93	3,8	5,4
**SF4**													
	**IL-1β**	**IFNγ**	**IFN-α2**	**TNFα**	**MCP-1**	**IL-6**	**IL-8**	**IL-10**	**IL-12p70**	**IL-17**	**IL-18**	**IL-23**	**IL-33**
**Lower range**	<1.17	<0.40	<0.724	<2.96	<2.57	<0.41	<0.70	<0,65	<0.43	<0.101	<2.29	<2.93	<2.8
**CD14-CD163-DCs**	<1,17	0,53	<0,724	<2,96	<2,57	<0,41	1004,76	1,07	1,09	0,11	10,93	<2,93	9,6
**CD14+CD163-DCs**	7,69	0,66	2,22	6,69	8,92	37,86	12000	18,78	2,35	0,2	20,6	6,28	7,5
**CD14+CD163+DCs**	97,92	0,68	2,71	39,14	209,21	807,78	12000	179,78	2,28	0,22	22,62	15,63	7,8
**SF5**													
	**IL-1β**	**IFNγ**		**TNFα**	**MCP-1**	**IL-6**	**IL-8**	**IL-10**	**IL-12p70**	**IL-17**	**IL-18**	**IL-23**	**IL-33**
**Lower range**	<1.17	<0.40	<0.724	<2.96	<2.57	<0.41	<0.70	<0,65	<0.43	<0.101	<2.29	<2.93	<2.8
**CD14-CD163-DCs**	3,31	<0,40	<0,724	<2,96	4,85	<0,41	1554,38	1,03	1,31	<0,101	15,67	<2,93	3,6
**CD14+CD163-DCs**	<1,17	<0,40	<0,724	<2,96	14,31	10,61	<0,70	<0,65	2883,55	<0,101	48,97	27,53	22,9
**CD14+CD163+DCs**	6,27	0,68	<0,724	6,74	6,75	17,24	6517,15	15,46	2	0,132	19,54	<2,93	5,5
